# Regular logarithmic connections

**DOI:** 10.1007/s00208-024-03047-9

**Published:** 2024-12-03

**Authors:** Piotr Achinger

**Affiliations:** Institute of Mathematics, Polish Academy of Sciences, ul. Śniadeckich 8, 00-656 Warsaw, Poland

## Abstract

We introduce the notion of a regular integrable connection on a smooth log scheme over $\textbf{C}$ and construct an equivalence between the category of such connections and the category of integrable connections on its analytification, compatible with de Rham cohomology. This extends the work of Deligne (when the log structure is trivial), and combined with the work of Ogus yields a topological description of the category of regular connections in terms of certain constructible sheaves on the Kato–Nakayama space. The key ingredients are the notion of a canonical extension in this context and the existence of good compactifications of log schemes obtained recently by Włodarczyk.

## Introduction

The notion of a regular connection was introduced by Deligne [6] in order to describe the category of complex representations of the topological fundamental group of a smooth scheme *X* over $\textbf{C}$ in an algebraic way. Denote by $\textrm{MIC}(X/\textbf{C})$ the category of coherent -modules *E* endowed with an integrable connection $\nabla :E\rightarrow E\otimes \Omega ^1_{X/\textbf{C}}$ (such an *E* is locally free). We say that *E* is *regular* if its (multi-valued) holomorphic horizontal sections have moderate growth at infinity. Deligne gave several useful characterizations of regular connections, relating them to the classical notion of Fuchsian singularities of differential equations, and proved that the functor sending *E* to its analytification $E_\textrm{an}$ induces an equivalence of categoriesbetween the category of regular connections on *X* and the category of integrable connections on the analytification $X_\textrm{an}$, which in turn is equivalent to the category $\textrm{LocSys}_\textbf{C}(X_\textrm{an})$ of complex local systems on $X_\textrm{an}$ via the functor sending *E* to its sheaf $E^\nabla$ of horizontal sections. The correspondence is compatible with cohomology in the sense that

$$\begin{aligned} H^*_\textrm{dR}(X, E) \, \simeq \, H^*_\textrm{dR}(X_\textrm{an}, E_\textrm{an})\, \simeq \, H^*(X_\textrm{an}, E_\textrm{an}^\nabla ) \,\, (Comparison Theorem \,\text {[6, II 6.2]}) \end{aligned}$$

This equivalence is often called the *Riemann–Hilbert correspondence*, later extended to an equivalence between regular holonomic *D*-modules on *X* and perverse sheaves on $X_\textrm{an}$ by Kashiwara [13] and Mebkhout [23]. The most important examples of regular connections are those coming from geometry, i.e. Gauss–Manin connections or Picard–Fuchs equations. Deligne proved that for a morphism $f:Y\rightarrow X$ which locally on *X* admits a relative simple normal crossings compactification, and a regular connection *E* on *Y*, the higher direct images $R^n f_* (E\otimes \Omega ^\bullet _{Y/X})$ with the Gauss–Manin connection are regular (*Regularity Theorem* [6, II 7.9]).

A pivotal role in the proofs of the above results is played by the notion of a logarithmic connection. An *snc pair* (*X*, *D*) consists of a smooth scheme *X* over $\textbf{C}$ and a simple normal crossings divisor $D\subseteq X$. An *(integrable) logarithmic connection* on (*X*, *D*) is a coherent -module *E* together with an integrable connection $E\rightarrow E\otimes \Omega _{X/\textbf{C}}(\log D)$ where $\Omega _{X/\textbf{C}}(\log D)$ is the sheaf of differentials with logarithmic poles along *D*. Such an *E* might not be locally free in general. As it turns out, the notion of regularity can be phrased in terms of logarithmic connections: for *X* smooth over $\textbf{C}$, an object *E* of $\textrm{MIC}(X/\textbf{C})$ is regular if and only if for every open $U\subseteq X$ and every compactification $U\hookrightarrow \overline{U}$ such that $(\overline{U}, D=\overline{U}\setminus U)$ is an snc pair, the restriction $E|_U$ extends to a logarithmic connection on $(\overline{U}, D)$. Such extensions are not unique, but it is possible to enumerate them, and a key idea in Deligne’s work ([6, Chap. II, Proposition 5.4], inspired by Manin [22]) is that one can single out a locally free *canonical extension* $\overline{E}$, the unique one in which the eigenvalues of the residue maps along the components of *D* (roughly, chosen logarithms of the local monodromy around *D*) belong to the set $\{0\le \textrm{Re}(z)<1\}$.

Logarithmic connections fit very well into the framework of logarithmic geometry [14]. An snc pair $(\underline{X}, D)$ corresponds to the log scheme  where  is the sheaf of regular functions on *X* invertible away from *D*; $\underline{X}$ is the “underlying scheme” of *X*. Every log scheme *X* has a sheaf of differentials $\Omega ^1_{X/\textbf{C}}$, and it is possible to define the category $\textrm{MIC}(X/\textbf{C})$ of coherent -modules endowed with a logarithmic connection. For an snc pair $(\underline{X}, D)$, the corresponding log scheme *X* is smooth in the sense of log geometry, we have $\Omega ^1_{X/\textbf{C}}\simeq \Omega ^1_{\underline{X}}(\log D)$, and integrable connections on *X* are precisely the integrable logarithmic connections on $(\underline{X}, D)$ as defined in the previous paragraph.

The goal of this article is to extend Deligne’s results to smooth logarithmic schemes over $\textbf{C}$. Since logarithmic connections are intimately tied to the notion of regularity, it is natural to treat them on the same footing as connections without poles. Given the interest in analytic logarithmic connections [12, 15, 27], it is a bit surprising that such a theory has not been developed earlier. That said, my main reason for doing so is the forthcoming work [1], in which I aim to give analogous results for “complex rigid-analytic spaces,” with applications to Hodge theory in mind. A typical space of interest in this context is the special fiber of a semistable formal scheme over $\textbf{C}[\![t]\!]$, endowed with the induced log structure. Since such log schemes do not arise from snc pairs $(\underline{X}, D)$, they are not captured by the classical theory, which corresponds to log schemes with *trivial* log structure.

Before going into the details of log geometry, let us explain how our definition of a regular logarithmic connection works for an snc pair $(\underline{X}, D)$. Since the log structure is nontrivial if $D\ne 0$, this case is not covered by Deligne’s work. For an integrable logarithmic connection $(E, \nabla )$ on $(\underline{X}, D)$ to be regular, it is insufficient to require that its restriction to $X\setminus D$ be regular (in Deligne’s sense), as *E* might be supported on *D*. To impose conditions along *D* as well, we proceed as follows. For every stratum *Z* of the stratification of *X* induced by the components of *D*, the connection $\nabla$ induces a map $\nabla |_Z:E|_Z\rightarrow E|_Z\otimes \Omega ^1_{X/\textbf{C}}(\log D)|_Z$. A local choice of equations of *D* along *Z* induces an isomorphismwhich allows us to deduce from $\nabla |_Z$ an integrable connection $E|_Z\rightarrow E|_Z\otimes \Omega ^1_{Z/\textbf{C}}$. While this connection depends on the choices made, its being regular does not, and we will say that *E* is *regular* if these connections are regular, for all *Z*.

We now turn to stating the general results. In order to gain some extra flexibility, we deal with “idealized smooth” log schemes over $\textbf{C}$, which look locally like the vanishing set of a monomial ideal in a toric variety (Definition 2.1). This class contains log schemes coming from snc pairs as well as special fibers of semistable formal schemes. In §2.3, for such a log scheme *X*, we construct a morphism

$$\begin{aligned} \pi :X^\#\longrightarrow X \end{aligned}$$

from a smooth scheme $X^\#$ (the disjoint union of certain torsors under tori over the log strata of *X*), and a canonical “splitting” $\varepsilon _\textrm{univ}$ of the log structure pulled back from *X*. The splitting $\varepsilon _\textrm{univ}$ behaves as if it were a section of the map from $X^\#$ to its underlying scheme $\underline{X}^\#$, even though no such section exists,^1^ and in particular it allows one to turn log connections into classical connections by means of a “pull-back” functor

$$\begin{aligned} \varepsilon ^\circledast _\textrm{univ}:\textrm{MIC}(X^\#/\textbf{C}) \longrightarrow \textrm{MIC}(\underline{X}^\#/\textbf{C}). \end{aligned}$$

### Definition 1.1

(See Definition 4.1) An object *E* of $\textrm{MIC}(X/\textbf{C})$ is *regular* if the object $\varepsilon ^\circledast _\textrm{univ}(\pi ^* E)$ of $\textrm{MIC}(\underline{X}^\#/\textbf{C})$ is regular (in Deligne’s sense). We denote by $\textrm{MIC}_\textrm{reg}(X/\textbf{C})$ the full subcategory of $\textrm{MIC}(X/\textbf{C})$ consisting of regular objects.

Our notion of regularity relies on Deligne’s, which makes many of the proofs easier. We give some familiar-looking characterizations of regularity including a “cut by curves” criterion. However, in setting up the theory, some extra difficulties have to be overcome: for example, as the functor $\varepsilon ^\circledast _\textrm{univ}(\pi ^* (-))$ is not exact and logarithmic connections might not be locally free, it is not obvious that subobjects of regular objects are regular. It is also not completely trivial to show that every connection on a proper *X* is regular, as $X^\#$ will typically not be proper.

The main results of this paper can be summarized as follows.

### Theorem 1.2

Let *X* be an idealized smooth log scheme over $\textbf{C}$.

- (*Existence Theorem* 4.14) The analytification functor $E\mapsto E_\textrm{an}$ induces an equivalence of categories
- (*Comparison Theorem* 4.17) Let *E* be an object of $\textrm{MIC}_\textrm{reg}(X/\textbf{C})$. Then
  $$\begin{aligned} H^*_\textrm{dR}(X, E)\simeq H^*_\textrm{dR}(X_\textrm{an}, E_\textrm{an}). \end{aligned}$$
- (*Regularity Theorem* 4.22) Let $f:Y\rightarrow X$ be a proper, smooth, and exact morphism and let *E* be an object of $\textrm{MIC}_\textrm{reg}(Y/\textbf{C})$. Assume that Conjecture 4.21 (a form of semistable reduction) holds. Then $R^n f_*(E\otimes \Omega ^\bullet _{Y/X})$ are regular for all $n\ge 0$.

In [27], Ogus described the category $\textrm{MIC}(X_\textrm{an}/\textbf{C})$ of analytic log integrable connections in terms of topological data, extending the earlier work [12, 15] in the case of (quasi-)unipotent local monodromy. More precisely, he constructed an equivalence between the category $\textrm{MIC}(X/\textbf{C})$ of integrable connections on an idealized smooth log complex analytic space *X* and the category *L*(*X*) of certain constructible sheaves on the Kato–Nakayama space $X_\textrm{log}$, compatible with cohomology (see Definition 3.11 and Theorem 3.12). Combined with Ogus’ results, Theorem 1.2 shows that we have an equivalence of categories

$$\begin{aligned} \textrm{MIC}_\textrm{reg}(X/\textbf{C}) \simeq L(X_\textrm{an}) \end{aligned}$$

compatible with cohomology. In §5, we use it to describe the category $\textrm{LocSys}_\textbf{C}(X_\textrm{log})$ of complex local systems on $X_\textrm{log}$ in terms of regular connections on *X*, assuming that stalks of the sheaf  are free.

As in [6], the proof of the Existence Theorem relies on the ability to extend an analytic connection to a compactification. In other words, we need to construct “canonical extensions” in the logarithmic context. Suitable compactifications of log schemes have only recently been constructed by Włodarczyk [30]. We call an open immersion $j:X\hookrightarrow \overline{X}$ of log schemes a *good compactification* (Definition 2.3) if $\overline{X}$ is proper and if étale locally the map *j* looks like the inclusion

$$\begin{aligned} {{\,\textrm{Spec}\,}}(\textbf{C}[P\times \textbf{Z}^r]/(K)) \hookrightarrow {{\,\textrm{Spec}\,}}(\textbf{C}[P\times \textbf{N}^r]/(K)) \end{aligned}$$

for some $r\ge 0$, a monoid *P*, and an ideal $K\subseteq P$. Then locally, every idealized smooth log scheme over $\textbf{C}$ admits a good compactification (Theorem 2.5). The above special form of the embedding (the fact that the “extra monoids at infinity” are free) enables us to define the exponents at infinity (analogues of the *negatives* of the eigenvalues of the residue maps) of an object $\overline{E}$ of $\textrm{MIC}(\overline{X}/\textbf{C})$. We then show (Theorem 3.17) that every object of $\textrm{MIC}(X_\textrm{an}/\textbf{C})$ admits a unique extension to an object of $\textrm{MIC}(\overline{X}/\textbf{C})$ whose exponents at infinity lie in $\{-1< \textrm{Re}(z)\le 0\}$. The proof relies on Ogus’ equivalence between $\textrm{MIC}(X_\textrm{an}/\textbf{C})$ and $L(X_\textrm{an})$, which allows us to reduce the question to graded modules over monoid algebras.

The definition of a regular logarithmic connection extends naturally to holonomic logarithmic *D*-modules in the sense of Koppensteiner–Talpo [17, 18], see Remark 4.13. This direction may deserve further study.

## Preliminaries

### Idealized smooth log schemes

We recall some terminology regarding (commutative) monoids and log schemes. For a monoid *P* (written additively), we write $P\rightarrow P^\textrm{gp}$ for its initial homomorphism into a group, and $P^\times \subseteq P$ for the largest subgroup of *P*. We say that *P* is *integral* if $P\rightarrow P^\textrm{gp}$ is injective. We write $\overline{P}=P/P^\times$ (quotient of the group action of $P^\times$) and say that *P* is *sharp* if $\overline{P}=P$. We say that *P* is *saturated* if it is integral and for every $x\in P^\textrm{gp}$ and $n\ge 1$ such that $nx\in \textrm{im}(P\rightarrow P^\textrm{gp})$ we have $x\in \textrm{im}(P\rightarrow P^\textrm{gp})$. A monoid *P* is *fs* (fine and saturated) if it is finitely generated and saturated. We note that if *P* is fs then $\overline{P}^\textrm{gp}$ is a free abelian group. An *ideal* of a monoid *P* is a subset $K\subseteq P$ such that $P+K=K$; it is *prime* if $x+y\in K$ implies $x\in K$ or $y\in K$. For an ideal *K* of *P* we write $\sqrt{K}$ for the ideal of elements $x\in P$ such that $nx\in K$ for some $n\ge 1$. A *face* of *P* is a submonoid $F\subseteq P$ such that $x+y\in F$ implies $x,y\in F$ (equivalently, $P\setminus F$ is a prime ideal). For a face *F* of *P*, we write $P_F = P + F^\textrm{gp}\subseteq P$ for the localization of *P* at *F*, and *P*/*F* for the quotient $P_F/F^\textrm{gp}$.

For a log scheme *X* we write $\underline{X}$ for its underlying scheme and  for its log structure, where  is written additively. We write  for the sheaf . A morphism of log schemes $f:X\rightarrow Y$ is *strict* if the map of log structures  is an isomorphism. For a ring *R*, a monoid *P*, and a homomorphism $P\rightarrow R$ (with addition on *P* and multiplication on *R*), we write ${{\,\textrm{Spec}\,}}(P\rightarrow R)$ for the scheme ${{\,\textrm{Spec}\,}}(R)$ endowed with the log structure associated to the map $P\rightarrow R$. We set $\textbf{A}_P = {{\,\textrm{Spec}\,}}(P\rightarrow \textbf{Z}[P])$; later on, when we work over $\textbf{C}$, we shall write $\textbf{A}_P$ for ${{\,\textrm{Spec}\,}}(P\rightarrow \textbf{C}[P])$. A log scheme *X* is *fs* if étale locally on $\underline{X}$ there exists an fs monoid *P* and a strict morphism $X\rightarrow \textbf{A}_P$. A morphism of log schemes $Y\rightarrow X$ is *strict étale* if it is both strict and étale, or equivalently if it is strict and the underlying morphism of schemes $\underline{Y}\rightarrow \underline{X}$ is étale.

#### Definition 2.1

- Let *P* be an fs monoid and let $K\subseteq P$ be an ideal. We set
  $$\begin{aligned} \textbf{A}_{P,K} = {{\,\textrm{Spec}\,}}(P\rightarrow \textbf{C}[P]/(K)) \end{aligned}$$
  for the scheme cut out by *K* in $\textbf{A}_P = {{\,\textrm{Spec}\,}}(P\rightarrow \textbf{C}[P])$ endowed with the induced log structure.
- Let *X* be an fs log scheme over $\textbf{C}$. We say that *X* is *smooth* if étale locally on $\underline{X}$ there exists an fs monoid *P* and a strict étale morphism $X\rightarrow \textbf{A}_{P}$. We say that *X* is *idealized smooth* if étale locally on $\underline{X}$ there exist an fs monoid *P*, an ideal $K\subseteq P$, and a strict étale morphism $X\rightarrow \textbf{A}_{P,K}$.

We make the analogous definition for fs log complex analytic spaces, using “locally” instead of “étale locally’. Then if *X* is an idealized smooth log scheme over $\textbf{C}$, then its analytification $X_\textrm{an}$ is an idealized smooth log complex analytic space. Note that if *X* is smooth, then  is the log structure induced by the largest open subset $X^\circ \subseteq X$ on which  is trivial. In particular, the map  is injective.

The following lemma often allows one to reduce to the case when the ideal *K* is trivial.

#### Lemma 2.2

Let *X* be an idealized smooth log scheme over $\textbf{C}$. Then étale locally on $\underline{X}$ there exist an fs monoid *P*, an ideal $K\subseteq P$ and a cartesian squarewhere $\textbf{A}_{P,K}\rightarrow \textbf{A}_P$ is the natural strict closed immersion and where the vertical maps are strict étale. In particular, *Y* is smooth over $\textbf{C}$ and *X* is cut out by a coherent ideal in .

#### Proof

We may assume that *X* is affine and that it admits a strict étale map $X\rightarrow \textbf{A}_{P,K}$ for *P* and *K* as in the statement. Write $A=\textbf{C}[P]$, $I=K\cdot A$, $\overline{A} = A/I$, and $X={{\,\textrm{Spec}\,}}(\overline{B})$. Thus $\overline{B}$ is an étale $\overline{A}$-algebra. By Artin approximation [8, Théorème 3] (which in the case of étale morphisms can be proved directly lifting the presentation in [29, Tag 03PC](3)), there exists an étale *A*-algebra $A'$ with $A'/IA' \simeq \overline{A}$ and an étale $A'$-algebra $B'$ such that $\overline{B} \simeq B'/IB'$ over *A*. Set $Y = {{\,\textrm{Spec}\,}}(B')$ and endow it with the log structure induced by the étale map $Y\rightarrow {{\,\textrm{Spec}\,}}(A) = \textbf{A}_P$. $\square$

### Compactifications of log schemes

#### Definition 2.3

**(Good embedding)** Let *X* be an idealized smooth log scheme over $\textbf{C}$ or an idealized smooth log complex analytic space.

- A *good embedding* is an open immersion $j:X\hookrightarrow \overline{X}$ which is locally isomorphic to the inclusion
  $$\begin{aligned} \textbf{A}_{P, K}\times \textbf{G}_m^r \hookrightarrow \textbf{A}_{P, K} \times \textbf{A}^r \end{aligned}$$
  for some $r\ge 0$, some fs monoid *P*, and some ideal $K\subseteq P$.
- For a good embedding, we denote by  the kernel of . It is a sheaf of faces of  whose stalks are free monoids, which we call the *residual face* of the good embedding $X\hookrightarrow \overline{X}$.
- A *good compactification* of *X* is a good embedding $j:X\hookrightarrow \overline{X}$ where *X* is proper.

#### Warning 2.4

If *X* is smooth (not just idealized smooth), and if $j:X\hookrightarrow \overline{X}$ is a good embedding, then . Indeed, the above description shows that the log structure is everywhere nontrivial outside *X*, i.e. we have $\overline{X} ^\circ = X^\circ$ where $X^\circ$ denotes the largest open subset on which the log structure is trivial. Thereforewhere $u:X^\circ \rightarrow X$ and ${\bar{u}}:\overline{X} ^\circ \rightarrow \overline{X}$ are the inclusions. However, if *X* is not assumed smooth, then typically the push-forward log structure  will be pathological, and in particular not equal to .^2^

For a simple example, let $\overline{X} = {{\,\textrm{Spec}\,}}(\alpha :\textbf{N}\times \textbf{N}\rightarrow \textbf{C}[t])$ where $\alpha (1,0) = 0$ and $\alpha (0,1) = t$. Let $X = \overline{X} {\setminus } \{0\} = {{\,\textrm{Spec}\,}}(\beta :\textbf{N}\rightarrow \textbf{C}[t,t^{-1}])$ with $\beta (1) = 0$. One readily computes the stalk (for the étale topology) at zero:In particular,which is not a finitely generated monoid, and hence  is not fs. We see that if $\varepsilon$ is the global section of  corresponding to the generator of $\textbf{N}$, then $\varepsilon t^{-1}$ is a global local section of  which is not in the image of a global section of .

#### Theorem 2.5

Let *X* be an idealized smooth log scheme over $\textbf{C}$. Then there exists a strict étale cover $\{X_i\rightarrow X\}_{i\in I}$ and good compactifications $X_i\hookrightarrow \overline{X}_i$.

#### Proof

Assume first that *X* is smooth. We phrase the proof in the language of toroidal embeddings used in [30]: let $D\subseteq X$ be the divisor where the log structure of *X* is nontrivial. The question being étale local, we may assume that *X* is affine, that the toroidal embedding (*X*, *D*) is strict (i.e., the log structure admits a chart Zariski locally), and that *D* has a unique closed stratum. By [30, Example 2.2.7], such a toroidal embedding is extendable ( [30, Definition 2.2.2]). By [30, Theorem 2.2.14], there exists a compactification $j:X\rightarrow \overline{X}$ such that $(\overline{X}, \overline{D})$ is again a strict toroidal embedding, where $\overline{D}$ denotes the closure of *D* in $\overline{X}$, and such that the divisor $E = \overline{X}\setminus X$ has *simple normal crossings* with $\overline{D}$ ( [30, Definitions 2.1.13 and 2.1.15]). The latter means precisely that $(\overline{X}, \overline{D}+E)$ is a strict toroidal embedding which locally has the form

$$\begin{aligned} (\textbf{A}_P\times \textbf{A}^r, (\partial \textbf{A}_P) \times \textbf{A}^r + \textbf{A}_P \times (\partial \textbf{A}^r)), \end{aligned}$$

where $\partial \textbf{A}_P$ (resp. $\partial \textbf{A}^r$) denotes the complement of $\textbf{A}_{P^\textrm{gp}}$ (resp. $\textbf{G}_m^r$). This means that the log scheme corresponding to the toroidal embedding $(\overline{X}, \overline{D}+E)$ is a good compactification of *X*.

In the general case, by Lemma 2.2 we may assume that *X* admits an exact closed immersion $X\hookrightarrow Y$ where *Y* is smooth and *X* is cut out by a coherent ideal in . Localizing further, we may assume that *Y* admits a good compactification $Y\hookrightarrow \overline{Y}$. Let $\overline{X}$ be the scheme-theoretic closure of *X* in $\overline{Y}$, endowed with the log structure induced from $\overline{Y}$. Then $X\hookrightarrow \overline{X}$ is a good compactification as well. To see this, it is enough to observe that the scheme-theoretic closure of $\textbf{A}_{P,K}\times \textbf{G}_m^r$ in $\textbf{A}_P\times \textbf{A}^r$ equals $\textbf{A}_{P,K}\times \textbf{A}^r$. $\square$

### Splittings of log structures

The basic invariant of an fs log structure  on a scheme *X* is the constructible sheaf of monoids , giving rise to the *log stratification* of *X* on whose strata it is locally constant. In this section, we deal with the situation when  is locally constant, in which case the log structure on *X* is “locally split.”

The main takeaway point for the rest of the paper is that for an idealized smooth log scheme *X* over $\textbf{C}$, there exists a strict morphism $X^\#\rightarrow X$ where $X^\#$ admits a splitting of the log structure, universal for all strict maps $Y\rightarrow X$ with *Y* reduced. Moreover, the underlying scheme of $X^\#$ is smooth over $\textbf{C}$.

We treat the case of fs log schemes, but the assertions apply equally well to fs log complex analytic spaces, formal schemes, algebraic spaces &c., with the meaning of the word *locally* adjusted accordingly.

#### Definition 2.6

Let *X* be an fs log scheme.

- We say that *X* has *locally constant log structure* if the sheaf  is locally constant.
- We say that *X* is *hollow* [26, §2, Definition 4] if  for every geometric point *x*.
- A *splitting* of the log structure  is a homomorphism of sheaves of monoids  such that the composition  is the identity.

For an integral monoid *P*, splittings of the projection $P\rightarrow \overline{P}=P/P^*$ are in bijection with splittings of the associated map of groups $P^\textrm{gp}\rightarrow \overline{P} ^\textrm{gp}$. Therefore, if *X* is an fs log scheme which locally admits a splitting and such that  is locally constant (as we shall shortly see, the two assumptions turn out to be equivalent), then splittings on *X* correspond to splittings of the locally split exact sequence of sheavesand therefore form a torsor under . The sheaf $T_X$ is the sheaf of sections of the torus over *X* with character sheaf  (see [3, Exposé X, §5–7]).

#### Lemma 2.7

Let *X* be an fs log scheme.

- Let $\varepsilon$ be a splitting on *X*, and let *m* be a local section of . Then $\alpha (\varepsilon (m))$ is locally on *X* either a unit or nilpotent.^3^
- *X* has locally constant log structure if and only if  locally admits a splitting.
- If *X* is hollow, then *X* has locally constant log structure. Conversely, if *X* is reduced and has locally constant log structure, then *X* is hollow.
- Let *P* be an fs monoid and let $K\subseteq P$ be an ideal. Then $\textbf{A}_{P,K}$ has locally constant log structure if and only if $\sqrt{K} = P\setminus P^\times$, i.e. if every element *x* of *P* is either invertible or satisfies $nx \in K$ for some $n\ge 1$. It is hollow if and only if $K = P\setminus P^\times$, and in this case we have $\textbf{A}_{P,K} \simeq {{\,\textrm{Spec}\,}}(\overline{P}\rightarrow \textbf{C}[P^\times ])$ where the map sends $\overline{P}\setminus 0$ to 0.
- Suppose that *X* is idealized smooth over $\textbf{C}$. If *X* has locally constant log structure, then $\underline{X}_\textrm{red}$ is smooth over $\textbf{C}$. It *X* is hollow if and only if it is reduced.

#### Proof

(a) We may assume $X={{\,\textrm{Spec}\,}}(R)$ for a strictly henselian local ring *R*, with closed point *x*. Let  and let . Since nilpotent elements are the intersection of all prime ideals, we need to show that if $f\notin \mathfrak {p}$ for some prime ideal $\mathfrak {p}\subseteq R$, then *f* is a unit. Let $y\in X$ be the point corresponding to such a $\mathfrak {p}$. We look at the commutative squareLet  be the image of *s*. We have $\varepsilon (\overline{s}) = us$ for some . As $\alpha (s)=f\notin \mathfrak {p}$, i.e. the image of $\alpha (s)$ in  is a unit, the image of *s* in  is in , so $\overline{s}$ maps to 0 in . Therefore $\varepsilon (\overline{s})=us$ maps to 1 in , and *uf* maps to 1 in . But then $uf-1$ maps to zero in , and hence is non-invertible in , so *f* is invertible.

(b) Suppose that *X* is locally split. By the proof of (a), we see that if *x* specializes to *y*, then  is bijective. By [26, §2, Lemma 5],  is locally constant. Conversely, suppose that  is locally constant, and we want to produce a splitting locally on *X*. To this end, we pick a point *x* and we may assume that  is constant with value *P* and *X* admits a neat chart [28, Chapter II, Definition 2.3.1] at *x*, i.e. a chart . This chart defines a splitting .

(c) Follows from (a) and (b).

(d) The orbits of the action of the diagonalizable group $T = {{\,\textrm{Spec}\,}}(\textbf{C}[P^\textrm{gp}])$ on $\textbf{A}_{P,K}$ correspond to prime ideals of *P* which contain *K*. The log structure on $\textbf{A}_{P,K}$ is locally constant if and only if there is a single orbit, corresponding to the maximal prime ideal $P\setminus P^\times$. Since $\sqrt{K}$ is the intersection of all prime ideals containing it, the first assertion follows. If $\textbf{A}_{P,K}$ is hollow, every element of *K* defines a nilpotent function and hence is equal to zero, showing $K = \sqrt{K} = P\setminus P^\times$. Since $\overline{P}^\textrm{gp}$ is a free abelian group, we may write $P = \overline{P}\oplus P^\times$. Then

$$\begin{aligned} \textbf{A}_{P,K}= &   {{\,\textrm{Spec}\,}}(\overline{P}\oplus P^\times \rightarrow \textbf{C}[P]/(P\setminus P^\times )) \simeq {{\,\textrm{Spec}\,}}(\overline{P}\oplus P^\times \rightarrow \textbf{C}[P^\times ])\\\simeq &   {{\,\textrm{Spec}\,}}(\overline{P} \rightarrow \textbf{C}[P^\times ]). \end{aligned}$$

(e) Follows from (d). $$\square $$

Note that splittings of the log structure can be pulled back along strict morphisms. That is, if $f:Y\rightarrow X$ is a strict morphism of fs log schemes and $\varepsilon$ is a splitting of , we have a natural mapwhich is a splitting of . This construction gives rise to the following functors $\textrm{Sch}_{\underline{X}}^\textrm{op} \rightarrow \textrm{Sets}$:We have natural transformations forming a cartesian square of functorsWe shall shortly recognize $X^\flat$ as the “log stratification” of *X*. The space $X^\#$, forming a torus torsor over $X^\flat$, will play a key role in this paper. The spaces $\widehat{X}^\#$ and $\widehat{X}^\flat$ will not be used in what follows.

#### Remark 2.8

Let *X* be a scheme and let  be a morphism of log structures on *X*. We get a commutative diagram of sheaves of abelian groupsTherefore the right square is a pullback, and consequently for every splitting $\varepsilon$ of  there exists a unique splitting $\varepsilon '$ of  for which the squarecommutes. If $f:Y\rightarrow X$ is a map of log schemes and $\varepsilon _X$ and $\varepsilon _Y$ are splittings on *X* and *Y* respectively, we shall say that *f* is *compatible* with $\varepsilon _X$ and $\varepsilon _Y$ if the splitting of  induced by $\varepsilon _X$ by pullback is equal to the splitting of  induced by $\varepsilon _Y$ as above.

This discussion implies that the four functors we have introduced are functorial in the sense that a map $Y\rightarrow X$ of fs log schemes induces maps $Y^\#\rightarrow X^\#$, $Y^\flat \rightarrow X^\flat$ etc. Moreover, the map $Y^\#\rightarrow X^\#$ is compatible with the universal splittings.

#### Proposition 2.9

Let *X* be an fs log scheme.

- The functors $\widehat{X}^\#$ and $\widehat{X}^\flat$ are representable by formal schemes affine over *X*, and the map $\widehat{X}^\#\rightarrow \widehat{X}^\flat$ is smooth and affine.
- The functors $X^\#$ and $X^\flat$ are representable by schemes of finite presentation and affine over *X*. The map $X^\#\rightarrow X^\flat$ is smooth and affine (a torsor under a torus over $X^\flat$) and the map $X^\flat \rightarrow X$ is a universally bijective monomorphism (the disjoint union of a locally finite family of finitely presented locally closed subschemes of *X*).

#### Example 2.10

Let $$X = \textbf{A}^1 = {{\,\textrm{Spec}\,}}(\textbf{N}\xrightarrow {1\mapsto t} \textbf{C}[t])$$. Then

$$\begin{aligned} \widehat{X}^\flat&\quad \simeq \quad {{\,\textrm{Spec}\,}}(\textbf{C}[t,t^{-1}]) \quad \sqcup \quad {\text {Spf}}(\textbf{C}\llbracket t\rrbracket ), \\ \widehat{X}^\#&\quad \simeq \quad {{\,\textrm{Spec}\,}}(\textbf{C}[t,t^{-1}]) \quad \sqcup \quad {\text {Spf}}(\textbf{C}\llbracket t\rrbracket \langle y, y^{-1}\rangle ), \\ X^\flat&\quad \simeq \quad {{\,\textrm{Spec}\,}}(\textbf{C}[t, t^{-1}]) \quad \sqcup \quad {{\,\textrm{Spec}\,}}(\textbf{C}), \\ X^\#&\quad \simeq \quad {{\,\textrm{Spec}\,}}(\textbf{C}[t, t^{-1}]) \quad \sqcup \quad {{\,\textrm{Spec}\,}}(\textbf{C}[y, y^{-1}]). \end{aligned}$$

Here, ${{\,\textrm{Spec}\,}}(A)$ is treated as the formal spectrum of *A* equipped with the discrete topology, and $\textbf{C}\llbracket t\rrbracket \langle y, y^{-1}\rangle$ is the *t*-adic completion of $\textbf{C}[t, y, y^{-1}]$.

#### Example 2.11

Let $X = \textbf{A}_{P}$ for an fs monoid *P*. For a face *F* of *P*, we denote by $X_F\hookrightarrow X$ the strict locally closed immersion corresponding to the map

2.1 $$\begin{aligned} \textbf{C}[P] \longrightarrow \textbf{C}[F^\textrm{gp}], \qquad p \mapsto {\left\{ \begin{array}{ll} p &  \text {if}\, p\in F, \\ 0 &  \text {otherwise}. \end{array}\right. } \end{aligned}$$

Thus $\underline{X}_F \simeq \textbf{A}_{F^\textrm{gp}}$. As we shall prove shortly, we have

2.2 $$\begin{aligned} X^\#&\simeq \bigsqcup _F X_F\times \textbf{A}_{(P/F)^\textrm{gp}}, \end{aligned}$$

2.3 $$\begin{aligned} X^\flat&\simeq \bigsqcup _F X_F, \end{aligned}$$

the coproducts taken over all faces $F\subseteq P$. The map $X^\#\rightarrow X^\flat$ is the disjoint union of the projections $X_F\times \textbf{A}_{(P/F)^\textrm{gp}}\rightarrow X_F$. Note that as *P*/*F* is sharp, $(P/F)^\textrm{gp}$ is a free abelian group, so the short exact sequence $0\rightarrow F^\textrm{gp}\rightarrow P^\textrm{gp}\rightarrow (P/F)^\textrm{gp}\rightarrow 0$ splits. Therefore $\underline{X}^\#$ is non-canonically isomorphic to the disjoint union of copies of the torus $X^* = \textbf{A}_{P^\textrm{gp}}$ indexed by faces of the monoid *P*. However, the log structures on the components depend on *F*.

#### Proof of Proposition 2.9

We first deal with the case $X = \textbf{A}_P = {{\,\textrm{Spec}\,}}(P\rightarrow \textbf{Z}[P])$ and reduce to this case afterwards. We will use the notation of Example 2.11.

We handle $\widehat{X}^\flat$ first. For a face *F* of *P*, let $P_F = P - F \subseteq P^\textrm{gp}$ be the localization of *P* at *F*, and let $K_F = P_F \setminus F^\textrm{gp}$, which generates the kernel of (2.1). Let $\mathfrak {X}_{F}$ denote the formal spectrum of the $K_F$-adic completion of $\textbf{Z}[P_F]$. We claim that $\widehat{X}^\flat$ is represented by $\bigsqcup _F \mathfrak {X}_{F}$. Since on the image of $X_F\rightarrow X$ the sheaf  is constant with value *P*/*F*, the log structure on each $\mathfrak {X}_{F}$ is locally constant. Let $Y\rightarrow X$ be a map such that the induced log structure on *Y* is locally constant. In particular, locally on *Y*, the pull-back of every $p\in P$ is either nilpotent or a unit (Lemma 2.7a). Since *P* is finitely generated, working locally on *Y*, we may assume that every *p* is either nilpotent or a unit or *Y*. Let $F\subseteq P$ be the set of $p\in P$ such that *p* is a unit on *Y*. Clearly, *F* is a face of *P*, the map  factors through $P_F$ and sends a power of $K_F$ to zero. Therefore $Y\rightarrow X$ factors uniquely through $\mathfrak {X}_{F}$.

The same proof shows that if $Y\rightarrow \textbf{A}_P$ is such that the induced log structure on *Y* is hollow, then the nilpotent functions above have to vanish, forcing the map to factor through ${{\,\textrm{Spec}\,}}(\textbf{Z}[P_F]/(K_F)) = X_F$. Therefore $X^\flat$ is represented by the disjoint union of $X_F$ taken over all faces *F* of *P*, and we proved (2.3).

Turning to $X^\#$ and $\widehat{X}^\#$, we need to show that if *X* has locally constant log structure to begin with, then the functor $\widehat{X}^\#$ is representable by a smooth scheme over *X*. But, the discussion preceding Lemma 2.7 shows that it is a torsor under the torus  and hence is representable (e.g. by [24, Theorem 4.3(a)]). A more direct analysis shows that in the case $X = {{\,\textrm{Spec}\,}}(P\rightarrow \textbf{Z}[F^\textrm{gp}])$ as in Example 2.11, we have . This gives the isomorphism (2.2).

Finally, we deal with the general case. Suppose first that $X'\rightarrow X$ is a strict étale surjection and that the statement holds for $X'$. Since the formation of the four functors $\widehat{X}^\#$, $\widehat{X}^\flat$, $X^\#$, $X^\flat$ commutes with strict base change, we deduce that they are representable by (formal) algebraic spaces over *X* with the required properties, and it remains to show that they are in fact (formal) schemes. However, $X^\flat \rightarrow X$ is affine, and hence $X^\flat$ is a scheme by étale descent for quasi-coherent sheaves. Similarly, $\widehat{X}^\flat$ is a formal scheme. Finally, $X^\#\rightarrow X^\flat$ and $\widehat{X}^\#\rightarrow \widehat{X}^\flat$ are affine as well, so $X^\#$ is a scheme and $\widehat{X}^\#$ is a formal scheme. Therefore the statement holds for *X*.

By the above, we may reduce to the case when *X* admits a strict morphism $X\rightarrow \textbf{A}_{P}$ for some monoid *P*, and since the statement holds for $\textbf{A}_P$ and the four functors commute with base change, the statement holds for *X* as well. $\square$

Although $X^\#$ and $X^\flat$ were defined (or described) as schemes over *X*, we endow them with the log structures pulled back from *X*. We denote by $\varepsilon _\textrm{univ}$ the canonical splitting of the log structure on $X^\#$.

#### Example 2.12

Let $X = {{\,\textrm{Spec}\,}}(P\rightarrow \textbf{C})$ be the log point associated to the sharp fs monoid *P*. Then $X^\# = {{\,\textrm{Spec}\,}}(P\rightarrow \textbf{C}[P^\textrm{gp}])$ where $P\setminus P^\times$ maps to zero. This chart corresponds to an “obvious” splitting $\varepsilon$, and  with $\varepsilon$ mapping *p* to (*p*, 0). This is not the same as the universal splitting $\varepsilon _\textrm{univ}$, which maps *p* to (*p*, *p*).

Using the local picture (Example 2.11) we deduce the following.

#### Corollary 2.13

Let *X* be an idealized smooth log scheme over $\textbf{C}$. Then, the underlying schemes of $X^\#$ and $X^\flat$ are smooth schemes over $\textbf{C}$.

The following definition will be used in §4.4.

#### Definition 2.14

A quasi-compact morphism of fs log schemes $f:Y\rightarrow X$ is *log dominant* if the following conditions are satisfied:

1. *f* is log injective, i.e. the maps  are injective for all geometric points $\overline{y}\rightarrow Y$,
2. the restriction of *f* to every log stratum of *X* is dominant, or equivalently, if the map $Y^\flat \rightarrow X^\flat$ is dominant.

We note that for a log dominant morphism $Y\!\rightarrow \! X$, the induced morphism $Y^\#\!\rightarrow \!X^\#$ is a dominant morphism of schemes.

### Algebraic and analytic sections

Let *X* be a scheme locally of finite type over $\textbf{C}$, let $X_\textrm{an}$ be the associated complex analytic space, and let $u:X_\textrm{an}\rightarrow X$ be the canonical map of locally ringed spaces. For a quasi-coherent -module , we write  its pull-back  to $X_\textrm{an}$. The functor  is exact, and  is a coherent analytic sheaf if  is coherent. We say that an analytic section  is *algebraic* (see [6, II.2]) if it lies in the image of the (injective) mapThe following result establishes a useful criterion for algebraicity of analytic sections of coherent sheaves.

#### Proposition 2.15

Let *X* be a scheme locally of finite type over $\textbf{C}$ and let  be a coherent -module. Let $\{Z_i\}_{i\in I}$ be a locally finite family of locally closed subschemes of *X* such that the union of $|Z_i|$ equals |*X*|. For $n\ge 0$, we denote by $Z_i ^{(n)}$ the *n*-th order thickening of $Z_i$ in *X*. Let  be an analytic section of . If for every $i\in I$ and every $n\ge 0$, the restriction of *s* to $Z_i ^{(n)}$ is algebraic, then so is *s*.

In other words, if we set

$$\begin{aligned} \pi :X' = \bigsqcup _{i\in I} \bigsqcup _{n\ge 0} Z_i^{(n)}\longrightarrow X, \end{aligned}$$

the proposition asserts that the following square is cartesian.

#### Remark 2.16

As the example $X={{\,\textrm{Spec}\,}}(\textbf{C}[x,y]/(y^2))$, $Z=V(y)$, , $s=y\exp (x)$ shows, it is not enough to consider the restrictions to $Z_i$, but we need to restrict to all of their thickenings as well. The issue is that $\bigsqcup Z_i\rightarrow X$ is not flat (see Corollary 2.18 below). Of course neither is $X'\rightarrow X$, but effectively $X'$ approximates the formal scheme $\mathfrak {X}'$ which is the disjoint union of the formal completions $\mathfrak {Z}_i$ of *X* along $Z_i$, and $\mathfrak {X}'\rightarrow X$ is flat. Since we do not wish to make sense of $\mathfrak {X}'_\textrm{an}$ (a formal complex analytic space), we use the scheme $X'$ instead of the formal scheme $\mathfrak {X}'$. The flatness of $\mathfrak {X}'\rightarrow X$ is reflected in the use of the Artin–Rees lemma in the proof below.

#### Lemma 2.17

Let *X* be a scheme locally of finite type over $\textbf{C}$ and let  be an injection of quasi-coherent -modules. A section  is algebraic if and only if its image in  is.

#### Proof

The assertion to be proved is equivalent to the left square in the diagram below being cartesian.The rows being exact and the right vertical map being injective, the claim follows by a simple diagram chase. $\square$

#### Corollary 2.18

Let *X* be a scheme locally of finite type over $\textbf{C}$ and let  be a coherent -module. Let $f:X'\rightarrow X$ be a flat morphism of finite type whose image contains the associated points of . Then, a section  is algebraic if and only if its image in  is.

#### Proof

Under the given assumptions, the map  is injective. We have a mapobtained by adjunction from the analytification of the counit map . It fits inside a commutative diagramSince the map denoted $\eta$ in the above diagram is injective, the image of *s* in  is algebraic. It remains to invoke Lemma 2.17. $\square$

#### Proof of Proposition 2.15

The question is local, so we can assume that *X* is affine and that there exists a finite filtrationof  by coherent subsheaves whose graded pieces are of the formfor some integral closed subschemes $Y_i\subseteq X$ [4, Chap. 7, Ex. 18]. We will proceed by induction on the length *m* of this filtration.

Consider $m=1$, i.e.  for some closed integral subscheme $Y\subseteq X$. There exists an index *i* such that $Z_i\cap Y$ is a dense open subset of *Y*. We conclude by applying Corollary 2.18 to the open immersion $Z_i\cap Y\hookrightarrow Y$ and the sheaf .

For the induction step, it suffices to prove that ifis a short exact sequence of coherent -modules such that the assertion of the proposition holds for both  and , then it holds also for . Let  be the image of *s*. Then $\pi ^*_\textrm{an} s''$ is algebraic, and by the assumption on  so is $s''$. Since *X* is affine, the short exact sequence stays exact after taking global sections, and hence there exists an  whose image in  equals $s''$.

Let . Suppose that $\pi ^*_\textrm{an} s'$ is algebraic, then by the assumption on  so is $s'$. Therefore $s = s' + s_0$ is algebraic as well. It remains to show that $\pi ^*_\textrm{an} s'$ is algebraic. The issue is that  might not be injective, see Remark 2.16. Working with one $Z_i$ at a time and replacing *X* with a suitable affine open cover of an open containing $Z_i$, we may reduce to the situation of Lemma 2.19 below, whose application finishes the proof. $\square$

#### Lemma 2.19

Let *X* be an affine scheme of finite type over $\textbf{C}$ and let $Z\subseteq X$ be a closed subscheme cut out by an ideal . We denote by $Z^{(n)}$ the closed subscheme of *X* cut out by . Let  be an injective map between coherent -modules and let  be an analytic section of . Suppose that for all $n\ge 0$, the image of *s* in  is algebraic. Then the image of *s* in  is algebraic for all $n\ge 0$.

#### Proof

By the Artin–Rees lemma, there exists a $k\ge 0$ such that for all $n\ge k$ we haveThis yields the commutative diagram with exact rowswhich implies that the image of *s* in  is the image of an element ofand hence is algebraic by Lemma 2.17 applied to the inclusion . $\square$

## Logarithmic connections and canonical extensions

### Connections on log schemes with constant log structure

#### Definition 3.1

Let *X* be an idealized smooth log scheme over $\textbf{C}$ or an idealized smooth log complex analytic space. An *integrable connection* on *X* is the data of a coherent -module *E* together with an integrable logarithmic connection $\nabla :E\rightarrow E\otimes \Omega ^1_{X/\textbf{C}}$. We denote by $\textrm{MIC}(X/\textbf{C})$ the category of integrable connections and horizontal maps.

As in the case of smooth schemes *X* (with no log structure), the category $\textrm{MIC}(X/\textbf{C})$ is a $\textbf{C}$-linear abelian category endowed with a symmetric monoidal tensor product $E\otimes F$ and internal Hom, denoted $\underline{\textrm{Hom}}(E, F)$. Unlike the classical case, its objects are not locally free, it is not rigid as a tensor category, and the tensor product is not exact.

The de Rham complex $\Omega ^\bullet _{X/\textbf{C}}\otimes E$ and the de Rham cohomology $H^*_\textrm{dR}(X, E) = H^*(X, E\otimes \Omega ^\bullet _{X/\textbf{C}})$ are defined in the usual way. Here we use the symbol $H^*(X, K^\bullet )$ to denote (hyper)cohomology with respect to the étale topology on $\underline{X}$, which in the case of $E\otimes \Omega ^\bullet _{X/\textbf{C}}$ agrees with cohomology with respect to the Zariski topology on $\underline{X}$. We have the useful formula

$$\begin{aligned} {{\,\textrm{Hom}\,}}(E, F) = H^0_\textrm{dR}(X, \underline{\textrm{Hom}}(E, F)). \end{aligned}$$

A morphism $f:X'\rightarrow X$ induces a pull-back functor

$$\begin{aligned} f^*:\textrm{MIC}(X/\textbf{C})\rightarrow \textrm{MIC}(X'/\textbf{C}) \end{aligned}$$

which agrees with the module pullback on underlying sheaves, and for every object *E* of $\textrm{MIC}(X/\textbf{C})$ a map $f^* :H^*_\textrm{dR}(X, E)\rightarrow H^*_\textrm{dR}(X', f^* E)$. For an idealized smooth log scheme *X* over $\textbf{C}$ we have an analytification functor

$$\begin{aligned} E\mapsto E_\textrm{an}\quad :\quad \textrm{MIC}(X/\textbf{C})\rightarrow \textrm{MIC}(X_\textrm{an}/\textbf{C}), \end{aligned}$$

and a natural morphism $H^*_\textrm{dR}(X, E)\rightarrow H^*_\textrm{dR}(X_\textrm{an}, E_\textrm{an})$.

#### Lemma 3.2

Let *X* be a hollow idealized smooth log scheme over $\textbf{C}$ or log complex analytic space. Then, the following hold.

- The underlying scheme $\underline{X}$ is smooth.
- The map  sending a local section *m* to the image of $d\log (m)$ (part of the universal log derivation on *X* relative to $\underline{X}$) annihilates the subsheaf  and induces an isomorphism
- The following sequence is exact.

#### Proof

Since *X* locally admits a strict étale map $X\rightarrow \textbf{A}_{P,K}$ where $K=P{\setminus } P^\times$, we may assume $X=\textbf{A}_{P,P\setminus P^\times }$. Then $\underline{X}={{\,\textrm{Spec}\,}}(\textbf{C}[P^\times ])$ is smooth, and the short exact sequence in question takes the form$\square$

**Fig. 1:**
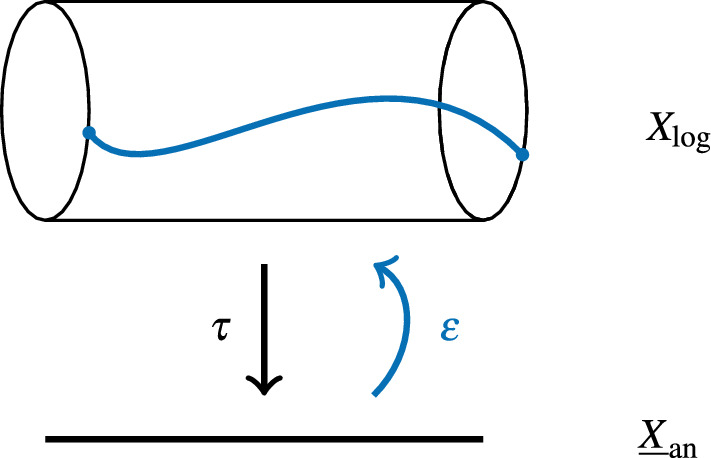
The Kato–Nakayama space of a log scheme *X* with a splitting $\varepsilon$

In this subsection, we shall explicate the category $\textrm{MIC}(X/\textbf{C})$ in the case when *X* is hollow and idealized smooth. For the sake of intuition, we note that the Betti realization of such an *X* is a torus bundle over $\underline{X}_\textrm{an}$. More precisely, the map $\tau :X_\textrm{log} \rightarrow \underline{X}_\textrm{an}$ is a torsor under the family of real tori . A splitting $\varepsilon$ of  provides a section of this torsor (see Fig. 1), and a pull-back functor $\varepsilon ^\circledast$ from local systems on $X_\textrm{log}$ to local systems on $\underline{X}_\textrm{an}$. We use the superscript $\circledast$ here to indicate that the functor is not induced by a map of log schemes $\underline{X}\rightarrow X$, though it behaves as if it was. The Künneth theorem allows one to identify local systems on $X_\textrm{log}$ with local systems on $\underline{X}_\textrm{an}$ endowed with an action of .

Proceeding by analogy, we shall now describe integrable connections on *X* in terms of integrable connections on $\underline{X}$ depending on the choice of a splitting $\varepsilon$.

#### Construction 3.3

Let *X* be a hollow idealized smooth log scheme over *k* and let $\varepsilon$ be a splitting on *X*. Although $\varepsilon$ (or rather the corresponding retraction ) does not come from a morphism of log schemes $\underline{X}\rightarrow X$, it induces a morphism

$$\begin{aligned} \varepsilon ^\circledast :\Omega ^1_{X/\textbf{C}}\longrightarrow \Omega ^1_{\underline{X}/\textbf{C}} \end{aligned}$$

as follows. Recall that giving a map  of -modules is equivalent to giving a log derivation with values in , which by definition is a pair of maps $(\partial , \partial ^\textrm{log})$ where  is a derivation and where  is a monoid homomorphism satisfying $\alpha (m)\partial ^\textrm{log}(m) = \partial (\alpha (m))$ for every local section *m* of . Let  be the map sending a local section *m* to $d\log (\varepsilon '(m))$ and let  be the universal derivation on $\underline{X}$. We claim that the pair $(d, d_\varepsilon ^\textrm{log})$ forms a log derivation on $X/\textbf{C}$. By definition, we need to verify the formula

$$\begin{aligned} \alpha (m)d_\varepsilon (m) = d\alpha (m) \end{aligned}$$

for a local section *m* of . By Lemma 2.7, $\alpha (m)$ is nilpotent if , so $\alpha (m)=0$ as $\underline{X}$ is reduced (see Lemma 2.7e), and the formula is satisfied in this case. For , the formula is obvious. We let $\varepsilon ^\circledast$ be the -linear map corresponding to this log derivation by the universal property of $\Omega ^1_{X/\textbf{C}}$.

Let  (Lemma 3.2). The map $\varepsilon ^\circledast$ splits the short exact sequence introduced in Lemma 3.2Using the induced decomposition , we see that the data of a logarithmic connection $E\rightarrow E\otimes \Omega ^1_{X/\textbf{C}}$ amounts to the data of a pair of maps $(\varepsilon ^\circledast (\nabla ), \rho _\nabla )$ defined aswhere $\varepsilon ^\circledast (\nabla )$ is a connection on $\underline{X}$ and where $\rho _\nabla$ is -linear.

#### Lemma 3.4

Let $\nabla :E\rightarrow E\otimes \Omega ^1_{X/\textbf{C}}$ be a logarithmic connection on an -module *E*. Then $\nabla$ is integrable if and only if the following conditions hold:

i. the connection $\varepsilon ^\circledast (\nabla ):E\rightarrow E\otimes \Omega ^1_{\underline{X}/\textbf{C}}$ is integrable,
ii. the map  is a -Higgs field,
iii. the map  is horizontal, where we endow  with the canonical connection satisfying $\nabla (m\otimes 1) = 0$, and  with the tensor product connection.

Before we give the (straightforward) proof, let us recall the notion of a Higgs field used in its statement. We will make use of the extra generality shortly.

#### Definition 3.5

Let  be a symmetric monoidal abelian category.

- For an object *F* of  and $r\ge 0$, the *r-th exterior power*  is the image of the antisymmetrization map $\sum _{\sigma \in S_r} (-1)^{\textrm{sign}(\sigma )}\sigma :F^{\otimes r}\rightarrow F^{\otimes r}$, see [5, §7, p. 165]. For $i,j\ge 0$, we denote by $\wedge$ the map
- Let *F* be an object of . An *F-Higgs object* of  is a pair $(E, \theta )$ where *E* is an object of  and $\theta :E\rightarrow E\otimes F$ is a morphism (called a *Higgs field*) satisfying $\theta ^1\circ \theta = 0$ where $\theta ^1$ is the map  A morphism of *F*-Higgs objects $(E',\theta ')\rightarrow (E,\theta )$ is a morphism $f:E'\rightarrow E$ for which the square  commutes. We denote by  the category of *F*-Higgs objects of ,^4^
- Let $(E, \theta )$ be an *F*-Higgs object of . The *Higgs complex* of $(E, \theta )$ is the complex  where the differential  is the composition

#### Proof of Lemma 3.4

Using the decomposition  induced by $\varepsilon ^\circledast$, we can describe the sequence $E\rightarrow E\otimes \Omega ^1_{X/C}\rightarrow E\otimes \Omega ^2_{X/\textbf{C}}$ as the totalization of the diagramwhere $\delta$ is the connection on , explicitly described as $\delta (e\otimes q) = s(\nabla (e)\otimes q)$ for $e\in E$ and  where  is the shuffle map. Therefore $\nabla ^1\circ \nabla = 0$ if and only if $\varepsilon ^\circledast (\nabla )^1 \circ \varepsilon ^\circledast (\nabla ) = 0$, $\rho _\nabla ^1\circ \rho _\nabla =0$, and the square in the diagram commutes. $\square$

#### Remarks 3.6

1. Suppose that  is constant with value $\textbf{N}^r$. The above shows that in the presence of a splitting $\varepsilon$ on *X*, the data of a log integrable connection on *X* is equivalent to the data of an integrable connection on $\underline{X}$ endowed with *r* commuting horizontal endomorphisms (the “residues”).
2. Let $f:Y\rightarrow X$ be a map between hollow idealized smooth log schemes over $\textbf{C}$ and let $\varepsilon _X$ and $\varepsilon _Y$ be splittings with which *f* is compatible (Remark 2.8). Then, we have an isomorphism of functors $\varepsilon ^\circledast _Y(f^*(-)) \simeq f^*(\varepsilon ^\circledast _X(-))$ from $\textrm{MIC}(X/\textbf{C})$ to $\textrm{MIC}(\underline{Y}/\textbf{C})$.
3. The following variant is quite useful in practice. Let $(\underline{X}, D)$ be an snc pair and let *X* be the associated log scheme. Let $D_1, \ldots , D_r$ be the components of *D*. For $I\subseteq \{1, \ldots , r\}$, let $Z=\bigcap _{i\in I} D_i$ be the closure of a stratum of *D* and let $\partial Z = Z\cap \bigcup _{i\notin I} D_i$. Then $(Z, \partial Z)$ is an snc pair, and we denote by $Z'$ the corresponding log scheme. We have a morphism of log schemes $Z\rightarrow Z'$ which is the identity on the underlying schemes, where the log structure on *Z* is induced by the one on *X*. This map étale locally admits a “splitting,” i.e. a splitting $\varepsilon$ of the map . In turn, the splitting $\varepsilon$ induces a functor $\varepsilon ^\circledast :\textrm{MIC}(Z/\textbf{C})\rightarrow \textrm{MIC}(Z'/\textbf{C})$ enjoying properties similar to those listed in Proposition 3.7 below. See [21, §3.3] for related calculations.
4. The dual map $(\varepsilon ^\circledast )^\vee :T_{\underline{X}/\textbf{C}}\rightarrow T_{X/\textbf{C}}$ defines a morphism of Lie algebroids, i.e. it is a morphism of $\textbf{C}$-Lie algebras on $\underline{X}$ (see [19, §2]) such that $u\circ (\varepsilon ^\circledast )^\vee$ is the identity where $u:T_{X/\textbf{C}}\rightarrow T_{\underline{X}/\textbf{C}}$ is the map induced by the map of log schemes $X\rightarrow \underline{X}$. In particular, we obtain a functor on the level of modules over those algebroids which agrees with $\varepsilon ^\circledast$.
5. The functor $\varepsilon ^\circledast :\textrm{MIC}(X/\textbf{C})\rightarrow \textrm{MIC}(\underline{X}/\textbf{C})$ extends to a functor  between the categories of coherent (log) *D*-modules [17, 18].

Summarizing the above discussion, we have proved:

#### Proposition 3.7

Let *X* be a hollow idealized smooth log scheme over $\textbf{C}$ and let $\varepsilon$ be a splitting on *X*. Then, the following assertions hold.

- The sheaf  is locally constant, $\underline{X}$ is smooth over $\textbf{C}$, and $\varepsilon$ induces a map
  $$\begin{aligned} \varepsilon ^\circledast :\Omega ^1_{X/\textbf{C}}\longrightarrow \Omega ^1_{\underline{X}/\textbf{C}} \end{aligned}$$
  splitting the exact sequence
- In turn, the map $\varepsilon ^\circledast$ induces a monoidal functor
  $$\begin{aligned} \varepsilon ^\circledast :\textrm{MIC}(X/\textbf{C}) \longrightarrow \textrm{MIC}(\underline{X}/\textbf{C}) \end{aligned}$$
  and an equivalence of categories
- The equivalence in (b) is compatible with cohomology in the following way. For an object $(E,\nabla )$ of $\textrm{MIC}(X/\textbf{C})$, we have a functorial isomorphism between the de Rham complex $E\otimes \Omega ^\bullet _{X/\textbf{C}}$ of *E* and the total complex of the double complex  whose *i*-th column is the de Rham complex of  and whose *j*-th row is the Higgs complex of *E* tensored with $\Omega ^j_{\underline{X}/\textbf{C}}$.

#### Corollary 3.8

Let *X* be a hollow idealized smooth log scheme over $\textbf{C}$. Then, every object of $\textrm{MIC}(X/\textbf{C})$ is locally free as an -module, and the category $\textrm{MIC}(X/\textbf{C})$ is a rigid tensor category [5, §2]. If *X* is connected, then for every point $x\in X(\textbf{C})$ and every splitting $\varepsilon$ on *x*, the functor

$$\begin{aligned} \omega =\varepsilon ^\circledast \circ x^*:\textrm{MIC}(X/\textbf{C})\longrightarrow \textrm{MIC}(\underline{x}/\textbf{C}) = \textrm{Vect}_\textbf{C}\end{aligned}$$

is an exact faithful $\textbf{C}$-linear tensor functor, making $(\textrm{MIC}(X/\textbf{C}), \omega )$ a Tannakian category.

#### Proof

Since *X* locally admits a splitting, for every object $(E, \nabla )$ of $\textrm{MIC}(X/\textbf{C})$, the -module *E* locally admits the structure of an object of $\textrm{MIC}(\underline{X}/\textbf{C})$ and hence is locally free. The rest follows. $\square$

The following straightforward lemma records the dependence of $\varepsilon ^\circledast (E)$ on the splitting $\varepsilon$.

#### Lemma 3.9

Let *X* be a hollow idealized smooth log scheme over $\textbf{C}$ and let $\varepsilon _i$ ($i=0,1$) be two splittings on *X*. We treat $\varepsilon _0/\varepsilon _1$ as a map . The composition  is additive and hence uniquely extends to an -linear map . Let $(E, \nabla )$ be an object of $\textrm{MIC}(X/\textbf{C})$. Then, the -linear map $\varepsilon ^\circledast _0(\nabla ) - \varepsilon ^\circledast _1(\nabla )$ equals the composition

Finally, we state a result for a general idealized smooth (non-hollow) log scheme *X*. Intuitively, it says that the “map” $\varepsilon _\textrm{univ}\circ \pi :\underline{X}^\#\rightarrow X$ is “unramified.”

#### Lemma 3.10

Let *X* be an idealized smooth log scheme over $\textbf{C}$ and let $\pi :X^\#\rightarrow X$ be the morphism constructed in §2.3, endowed with the universal splitting $\varepsilon _\textrm{univ}$. Then, the compositionis an isomorphism.

#### Proof

Since the log stratification $p:X^\flat \rightarrow X$ satisfies $p^*\Omega ^1_{X/\textbf{C}}\simeq \Omega ^1_{X^\flat /\textbf{C}}$, we may assume that *X* is hollow. Working locally, by Lemma 2.7(d) we may assume that $X={{\,\textrm{Spec}\,}}(P\rightarrow \textbf{C})\times \textbf{G}_m^n$ for some sharp fs monoid *P*, where $P\rightarrow \textbf{C}$ maps $P{\setminus } 0$ to 0. In this case, $X^\# = {{\,\textrm{Spec}\,}}(P\rightarrow \textbf{C}[P^\textrm{gp}])\times \textbf{G}_m^n$ where $P\rightarrow \textbf{C}[P^\textrm{gp}]$ maps $P\setminus 0$ to 0, and $\varepsilon _\textrm{univ}(p) = (p,p)$ (see Example 2.12). The rest follows by a direct calculation using the formula

$$\begin{aligned} \varepsilon ^\circledast _\textrm{univ}\pi ^*(d\log (p)) = d\log (p). \end{aligned}$$

$$\square $$

### Ogus’ Riemann–Hilbert correspondence

Ogus’ correspondence [27] describes the category $\textrm{MIC}(X/\textbf{C})$ of integrable connections on an idealized smooth log complex analytic space *X* in terms of certain constructible sheaves on the Kato–Nakayama space $X_\textrm{log}$.

At its kernel, it is a generalization of the following basic fact. A locally free log connection on the open unit disc can be put in the form  where $U\in M_{n\times n}(\textbf{C})$ is a constant matrix. The monodromy of the associated local system on the punctured disc is given by $T = \exp (-2\pi i U)$ [6, Chap. II, Lemme 1.17.1]. In order to recover the log connection from its monodromy, we need to describe the set of “logarithms” of the matrix *T*, which is in bijection with the set of $\textbf{C}$-gradings $\textbf{C}^n=\bigoplus _{\lambda \in \textbf{C}} V_\lambda$ stable under *U* and such that *U* has unique eigenvalue $\exp (-2\pi i\lambda )$ on $V_\lambda$ for every $\lambda \in \textbf{C}$, determined by the condition that *T* has unique eigenvalue $\lambda$ on $V_\lambda$.

#### Definition 3.11

([27, Definition 3.2.4]) Let *X* be an idealized smooth log complex analytic space. We set , and definewhere $\tau :X_\textrm{log}\rightarrow X$ is the Kato–Nakayama space of *X*. The inclusion  makes $\textbf{C}_X^\textrm{log}$ into a $\Lambda _X$-graded sheaf of rings on $X_\textrm{log}$. We also set  for $x\in X$.

We denote by *L*(*X*) the category (denoted $L^\Lambda _\textrm{coh}(\textbf{C}_X^\textrm{log})$ in [27]) of $\Lambda _X$-graded $\textbf{C}_X^\textrm{log}$-modules *V* satisfying the following conditions:

1. *V* is log constructible [27, Definition 3.2.3],
2. for every $x\in X_\textrm{log}$, the stalk $V_x$ is finitely generated over ,
3. for every log path [27, p. 704] from *x* to *y* in $X_\textrm{log}$, the cospecialization map
  $$\begin{aligned} \gamma _{x,y}^* :V_x \otimes _{\textbf{C}_{X,x}^\textrm{log}} \textbf{C}_{X,y}^\textrm{log} \longrightarrow V_y \end{aligned}$$
  is an isomorphism,
4. for every $x\in X_\textrm{log}$, every $\gamma \in I_{\tau (x)}$ and every $\lambda \in \Lambda _{X,x}$, the number $\exp \langle \lambda , \gamma \rangle$ is the unique eigenvalue of $\gamma :V_{x,\lambda }\rightarrow V_{x,\lambda }$.

#### Theorem 3.12

([27, Theorem 3.4.2]) Let *X* be an idealized smooth log complex analytic space.^5^ There is an equivalence of tensor categoriesfunctorial with respect to maps $Y\rightarrow X$ of idealized smooth log complex analytic spaces. Furthermore, there is a functorial (in both *E* and *X*) quasi-isomorphismwhere the subscript 0 denotes the zeroth graded piece.

#### Remark 3.13

(Functoriality of ) Functoriality of the equivalence  with respect to *X* means the following. Let $f:Y\rightarrow X$ be a map of idealized smooth log complex analytic spaces. The pullback functor

$$\begin{aligned} f^*:L(X)\longrightarrow L(Y) \end{aligned}$$

is induced by the module pullback functor with respect to the map of ringed spaces

$$\begin{aligned} (Y_\textrm{log}, \textbf{C}_Y^\textrm{log})\rightarrow (X_\textrm{log}, \textbf{C}_X^\textrm{log}), \end{aligned}$$

with $\Lambda _Y$-grading induced by the $\Lambda _X$ grading via the map $f^* \Lambda _X\rightarrow \Lambda _Y$. Then, functoriality means the existence of a natural isomorphism between the two compositions in the square below.3.1

#### Example 3.14

Let *P* be a sharp fs monoid and let $K\subseteq P$ be an ideal. Let *L*(*P*, *K*) be the category of finitely generated $P^\textrm{gp}\otimes \textbf{C}$-graded $\textbf{C}[P]/(K)$-modules $V = \bigoplus _{\lambda \in P^\textrm{gp}\otimes \textbf{C}}V_\lambda$ endowed with a homogeneous action of $\pi _1(P) = {{\,\textrm{Hom}\,}}(P^\textrm{gp}, \textbf{Z}(1))$ such that for every $\gamma \in \pi _1(P)$ and every $\lambda \in P^\textrm{gp}\otimes \textbf{C}$, the number $\exp \langle \lambda ,\gamma \rangle$ is the unique eigenvalue of $\gamma :V_\lambda \rightarrow V_\lambda$. Let $X = \textbf{A}_{P,K}^\textrm{an}$. Then by [27, Proposition 3.2.5] there is a tensor equivalence

$$\begin{aligned} L(X_\textrm{an}) \simeq L(-P, -K). \end{aligned}$$

#### Lemma 3.15

Let *P* be a sharp fs monoid and let $K\subseteq P$ be an ideal. Let $F\subseteq P$ be a face disjoint from *K* and let $P' = P_F$, $K' = K + P'$. Let $X = \textbf{A}_{P,K}^\textrm{an} \subseteq {{\,\textrm{Hom}\,}}(P, \textbf{C})$ and $X' = \textbf{A}_{P',K'}^\textrm{an} \subseteq X$. For a generating set *S* of *P* and a real number $\delta >0$, consider the following open subsets:

$$\begin{aligned} U_\delta&= \{ x\in X \,:\, |x(p)|<\delta \, \text {for}\, p\in S\}, \\ U'_\delta&= U_\delta \cap X' \end{aligned}$$

Then, there are tensor equivalences $L(U_\delta ) \simeq L(-P, -K)$ and $L(U'_\delta )\simeq L(-P', K')$ fitting inside a commutative square

#### Proof

We first show that for every face *F* of *P* disjoint from *K*, the inclusion of $U_\delta \cap X_F\hookrightarrow X_F$ is a homotopy equivalence. Here $X_F = \textbf{A}_{F^\textrm{gp}}$ is the stratum corresponding to *F*. To show this we may ignore the ideal *K*. We also note that $S\cap F$ is a generating set for *F*. Identifying *X* with ${{\,\textrm{Hom}\,}}(P, \textbf{C})$, we have

$$\begin{aligned} X_F&= \{x:P\rightarrow \textbf{C}\,:\, x^{-1}(\textbf{C}^\times ) = F \} \\&= {{\,\textrm{Hom}\,}}(F^\textrm{gp}, \textbf{C}^\times ) = {{\,\textrm{Hom}\,}}(F^\textrm{gp}, \textbf{R}_{>0}) \times {{\,\textrm{Hom}\,}}(F^\textrm{gp}, \textbf{S}^1),\\ X_F\cap U_\delta&= \{x:F^\textrm{gp}\rightarrow \textbf{C}^\times \,:\, |x(p)|<\delta \, \text {for}\, p\in S\cap F \} \\&= \{x:F^\textrm{gp}\rightarrow \textbf{R}_{>0} \,:\, x(p)<\delta \, \text {for}\, p\in S\cap F \} \times {{\,\textrm{Hom}\,}}(F^\textrm{gp}, \textbf{S}^1). \end{aligned}$$

It remains to observe that the set cut out by the equations $x(p)<\delta$ in the convex cone ${{\,\textrm{Hom}\,}}(F^\textrm{gp}, \textbf{R}_{>0})$ is convex and contains a neighborhood of zero, and hence is contractible, so that its inclusion into ${{\,\textrm{Hom}\,}}(F^\textrm{gp}, \textbf{R}_{>0})$ is a homotopy equivalence.

The following description of log constructible sheaves on $X^\textrm{log}$ where $X=\textbf{A}_{P,K}$ appears in [27, §3.2]. Such a sheaf corresponds to the data of a functorwhere  is the stalk of the corresponding sheaf at a prescribed point of $X_F^\textrm{log}$, and where the maps  for $F'\subseteq F$ correspond to the cospecialization maps. In these terms, the sheaf $\textbf{C}_X^\textrm{log}$ corresponds to the family of algebras $\textbf{C}[-P/F]/(-K)$, the sheaf of gradings $\Lambda _X$ corresponds to the family of groups $(P/F)^\textrm{gp}\otimes \textbf{C}$, and an object *V* of *L*(*X*) corresponds to the family of finitely generated $(P/F)^\textrm{gp}\otimes \textbf{C}$-graded $\textbf{C}[-P/F]/(-K)$-modules $V_F$ endowed with an action of $\pi _1(P)$ satisfying the suitable condition on eigenvalues, and such that the cospecialization map for the inclusion $0\subseteq F$ induces an isomorphism $V_F = V_0\otimes _{\textbf{C}[-P]/(-K)}\textbf{C}[-P/F]/(-K)$. It is therefore determined by the object $V_0\in L(-P,-K)$ (see the discussion following [27, Definition 3.2.4]), and so *L*(*X*) is equivalent to the category $L(-P,-K)$. We have just explained the proof of [27, Proposition 3.2.5] mentioned in Example 3.14 above.

The point of going into Ogus’ proof is that his argument for this description, employing the universal cover of $X^\textrm{log}$, applies equally well to any open subset $U\subseteq X^\textrm{an}$ with the property that for every face *F* of *P* disjoint from *F*, the preimage of $U\cap X_F$ in the universal cover of $X^\textrm{log}$ is contractible. In particular, by the result of the first paragraph, it applies to the sets $U_\delta$ and $U'_\delta$, and we obtain our assertion. $\square$

### Canonical extensions

In this section, we fix a good embedding $j:X\hookrightarrow \overline{X}$ of log complex analytic spaces (Definition 2.3). For $x\in \overline{X}$, we have a canonical isomorphismwhere  for some $s\ge 0$ depending on *x*. For an object *E* of $\textrm{MIC}(\overline{X}/\textbf{C})$, the generators $e_1, \ldots , e_s$ (well defined up to permutation) of  induce linear endomorphisms (“residues”)

$$\begin{aligned} \rho _i :E_x \longrightarrow E_x, \qquad E_x = E\otimes \kappa (x). \end{aligned}$$

We denote by $R_x\subseteq \textbf{C}$ the union of the set of negatives of the eigenvalues of $\rho _1, \ldots , \rho _s$ and call it the set of *exponents at infinity* of *E* at *x*.

#### Definition 3.16

Let $\sigma :\textbf{C}/\textbf{Z}\rightarrow \textbf{C}$ be a section of the projection $\textbf{C}\rightarrow \textbf{C}/\textbf{Z}$ and let $j:X\rightarrow \overline{X}$ be a good embedding. We say that an object $E\in \textrm{MIC}(\overline{X}/\textbf{C})$ is $\sigma$*-adapted* if:

1. for every $x\in \overline{X}$, the set $R_x$ of exponents at infinity of *E* at *x* is contained in $\textrm{im}(\sigma )$,
2. the map $E\rightarrow j_* j^* E$ is injective.

We denote by $\textrm{MIC}^\sigma (\overline{X}, X/\textbf{C})$ the full subcategory of $\textrm{MIC}(\overline{X}/\textbf{C})$ consisting of $\sigma$-adapted objects.

#### Theorem 3.17

Let $\sigma :\textbf{C}/\textbf{Z}\rightarrow \textbf{C}$ be a section of the projection $\textbf{C}\rightarrow \textbf{C}/\textbf{Z}$.

- The restriction functor induces an equivalence
- If $\sigma (0)=0$, then for every object *E* of $\textrm{MIC}^\sigma (\overline{X}, X/\textbf{C})$ the map
  $$\begin{aligned} j^* :H^*_\textrm{dR}(\overline{X}, E)\longrightarrow H^*_\textrm{dR}(X, j^* E) \end{aligned}$$
  is an isomorphism.
- An object *E* of $\textrm{MIC}^\sigma (\overline{X}, X/\textbf{C})$ is locally free (as an -module) if and only if its restriction to *X* is locally free.

Before proving the result, we show an auxiliary statement about monoids.

#### Lemma 3.18

Let *P* be a sharp fs monoid and let $Q = P\times \textbf{N}^r$ and $Q' = P\times \textbf{Z}^r$. Let $K\subseteq P$ be an ideal, and denote also by *K* the ideal it generates in *Q* and $Q'$. Denote by $L^\sigma (Q, K)$ the full subcategory of *L*(*Q*, *K*) (see Example 3.14) consisting of objects *V* such that

1. *V* is generated as a $\textbf{C}[Q]$-module by $\bigoplus _{\lambda \in (P^\textrm{gp}\otimes \textbf{C})\times \textrm{im}(\sigma )^r} V_\lambda$,
2. the map $V\rightarrow V' = V\otimes _{\textbf{C}[Q]} \textbf{C}[Q']$ is injective.

Then

- the functor $V\mapsto V' = V\otimes _{\textbf{C}[Q]} \textbf{C}[Q']$ induces an equivalence of categories
- an object *V* of $L^\sigma (Q)$ is free as a $\textbf{C}[Q]/(K)$-module if and only if $V'$ is free as a $\textbf{C}[Q']/(K)$-module.

#### Proof

Let $\Sigma = \{z\in \textbf{C}:\, z-\sigma (z) \in \textbf{N}\}$. For an object $V'$ of $L(Q', K)$, we set

$$\begin{aligned} V = \bigoplus _{\lambda \in (P^\textrm{gp}\otimes \textbf{C})\times \Sigma ^r} V'_\lambda . \end{aligned}$$

This is a $Q^\textrm{gp}\otimes \textbf{C}$-graded $\textbf{C}[Q]$-module with an action of $\pi _1(Q) = \pi _1(Q')$ satisfying the required condition on eigenvalues. We check that it is finitely generated by homogeneous elements lying in degrees in

$$\begin{aligned} (P^\textrm{gp}\otimes \textbf{C})\times \textrm{im}(\sigma )^r. \end{aligned}$$

Let $v'_1, \ldots , v'_r$ be homogeneous generators of $V'$ lying in degrees $\lambda '_i = (\eta _i, \lambda '_{i,1}, \ldots , \lambda '_{i,r})$. Let

$$\begin{aligned} v_i= &   x_1^{\sigma (\lambda '_{i,1})-\lambda '_{i,1}} \cdots x_r^{\sigma (\lambda '_{i,r})-\lambda '_{i,r}}v'_i \in V'_{\lambda _i}, \quad \lambda _i \\  = &   (\eta _i, \sigma (\lambda '_{i,1}), \ldots , \sigma (\lambda '_{i,r})) \in (P^\textrm{gp}\otimes \textbf{C})\times \textrm{im}(\sigma )^r, \end{aligned}$$

these elements generate *V*. Finally, we have $V' = V\otimes _{\textbf{C}[Q]}\textbf{C}[Q']$, so $V\rightarrow V\otimes _{\textbf{C}[Q]}\textbf{C}[Q']$ is injective. The construction $V'\mapsto V$ is functorial in $V'$ and produces a quasi-inverse to the given functor, showing (a).

To show (b), suppose that $V'$ is freely generated by the homogeneous elements $v'_i\in V'_{\lambda '_i}$. Then *V* is freely generated by the elements $v_i$. $\square$

#### Proof of Theorem 3.17

(a) The subcategory $\textrm{MIC}^\sigma (\overline{X},X/\textbf{C})$ of $\textrm{MIC}(\overline{X}/\textbf{C})$ is defined by a pointwise condition and hence is of local nature on $\overline{X}$. Localizing near a point $x\in \overline{X}$ and using the local form of a good embedding, we may assume that $\overline{X}$ is an open subset of the form $U_\delta$ of $\textbf{A}_{P\times \textbf{N}^r, K}$ appearing in Lemma 3.15, so that $X = U'_\delta$. Using that lemma combined with Theorem 3.12 and [27, Remark 2.1.3], we obtain a commutative square of categories and functorswhere $L^\sigma (P\times \textbf{N}^r, K)$ is as in Lemma 3.18. The bottom arrow is an equivalence by (a) of that lemma, and the assertion follows.

(b) Using the argument in (a), this follows from Lemma 3.18(b). $\square$

## Regular connections and the Riemann–Hilbert correspondence

### Regular connections

Let *X* be an idealized smooth log scheme over $\textbf{C}$, let $\pi :X^\#\rightarrow X$ be the morphism constructed in Proposition 2.9, and let $\varepsilon _\textrm{univ}$ be the universal splitting of the log structure . The log scheme $X^\#$ is idealized smooth and hollow, and so its underlying scheme is smooth (Lemma 3.2a). As in §3.1, we have a functor

$$\begin{aligned} \varepsilon ^\circledast _\textrm{univ} :\textrm{MIC}(X^\#/\textbf{C}) \longrightarrow \textrm{MIC}(\underline{X}^\#/\textbf{C}). \end{aligned}$$

#### Definition 4.1

With notation as above, we say that an object *E* of $\textrm{MIC}(X/\textbf{C})$ is *regular* if the object $\varepsilon ^\circledast _\textrm{univ}(\pi ^* E)$ of $\textrm{MIC}(\underline{X}^\#/\textbf{C})$ is regular in the classical sense [6, Chap. II, Définition 4.2]. We denote by $\textrm{MIC}_\textrm{reg}(X/\textbf{C})$ the full subcategory of $\textrm{MIC}(X/\textbf{C})$ consisting of the regular objects.

We note right away that if *X* has trivial log structure, then $X=X^\#=\underline{X}$ and an object *E* of $\textrm{MIC}(X/\textbf{C}) = \textrm{MIC}(\underline{X}/\textbf{C})$ is regular in the above sense if and only if it is regular in the classical sense.

The use of the universal splitting $\varepsilon ^\circledast _\textrm{univ}$ on $X^\#$ is nicely canonical. However, any splitting will do:

#### Lemma 4.2

Let *X* be a hollow idealized smooth log scheme over $\textbf{C}$ and let $\varepsilon _i$ ($i=0,1$) be two splittings on *X*. Let *E* be an object of $\textrm{MIC}(X/\textbf{C})$. Then $\varepsilon ^\circledast _0(E)$ is regular if and only if $\varepsilon ^\circledast _1(E)$ is regular.

#### Proof

We may assume that  is constant with value *P* and that $\underline{Y}$ admits a good compactification $\underline{Y}\hookrightarrow \overline{Y}$ (which in this case means the usual snc compactification). Write $\nabla _i = \varepsilon ^\circledast _i(\nabla )$ for the connection on $\varepsilon ^\circledast _i(E)$, and suppose that $\varepsilon ^\circledast _0(E)=(E,\nabla _0)$ is regular. Let $\overline{E}$ be the canonical extension of $(E, \nabla _0)$ to a connection on $\overline{Y}$ with logarithmic poles along $D = \overline{Y}\setminus \underline{Y}$. We claim that $\nabla _1$ extends to a logarithmic connection on $\overline{E}$ as well, showing that $\varepsilon ^\circledast _1(E)=(E, \nabla _1)$ is regular.

By Lemma 3.9, we have $\nabla _1 = \nabla _0 - (1\otimes \delta (\varepsilon _0, \varepsilon _1))\circ \rho _\nabla$. Pick a basis $p_1, \ldots , p_r$ of $P^\textrm{gp}$, and write . Then $d\log (f_i)$ is a section of $\Omega ^1_{\overline{Y}/\textbf{C}}(\log D)$. We explicate $\nabla _1$ as

$$\begin{aligned} \nabla _1 = \nabla _0 - \sum _i \rho _i \otimes d\log (f_i) \end{aligned}$$

where $\rho _i:E\rightarrow E$ is the residue map induced by $p_i$. By functoriality of the canonical extension, since the maps $\rho _i:E\rightarrow E$ are horizontal with respect to $\nabla _0$, they extend uniquely to morphisms $\overline{\rho }_i:\overline{E}\rightarrow \overline{E}$. Therefore $\nabla _1$ maps $\overline{E}$ into $\overline{E}\otimes \Omega ^1_{\overline{Y}/\textbf{C}}(\log D)$. $\square$

#### Lemma 4.3

Let *X* be an idealized smooth log scheme over $\textbf{C}$ and let *E* be an object of $\textrm{MIC}(X/\textbf{C})$. The following conditions are equivalent.

- *E* is regular,
- for every idealized smooth and hollow *Y* with a strict map $f:Y\rightarrow X$, and every splitting $\varepsilon$ of , the object $\varepsilon ^\circledast (f^* E)$ of $\textrm{MIC}(\underline{Y}/\textbf{C})$ is (classically) regular,
- there exists a log dominant (Definition 2.14) morphism $f:Y\rightarrow X$ with *Y* idealized smooth and hollow and a splitting $\varepsilon$ on *Y* such that $\varepsilon ^\circledast (f^* E)$ is (classically) regular.

#### Proof

Every $f:Y\rightarrow X$ as in (b) uniquely factors through a map ${\tilde{f}}:Y\rightarrow X^\#$ such that the splitting $\varepsilon$ is the preimage of the universal splitting $\varepsilon _\textrm{univ}$. Then

$$\begin{aligned} \varepsilon ^\circledast (f^* E) = \varepsilon ^\circledast ({\tilde{f}}^* \pi ^* E) = {\tilde{f}}^* \varepsilon ^\circledast _\textrm{univ}(\pi ^* E) \end{aligned}$$

which shows that (a) implies (b). Moreover, (b) implies (c).

It remains to show that (c) implies (a). We have a commutative square (see Remark 2.8)where the top map *g* is a dominant as a map of schemes. Denote by $\varepsilon _0$ and $\varepsilon _1$ the splittings on $Y^\#$ induced by $\varepsilon$ and $\varepsilon _\textrm{univ}$, respectively. By Lemma 4.2, we have

$$\begin{aligned} \varepsilon ^\circledast _0(g^* \pi ^* E)\, \text {is regular} \quad \Leftrightarrow \quad \varepsilon ^\circledast _1(g^* \pi ^* E)\, \text {is regular}. \end{aligned}$$

But $\varepsilon ^\circledast _0(g^* \pi ^* E) \simeq \varepsilon ^\circledast _0(\pi _Y^* f^* E) = \pi _Y^* (\varepsilon ^\circledast (f^* E))$, which is regular since $\varepsilon ^\circledast (f^* E)$ is. Therefore

$$\begin{aligned} \varepsilon ^\circledast _1(g^* \pi ^* E) \simeq g^* (\varepsilon ^\circledast _\textrm{univ}(\pi ^* E)) \end{aligned}$$

is regular, and hence so is $\varepsilon ^\circledast _\textrm{univ}(\pi ^* E)$ by [6, Chap. II, Proposition 4.6(iii)]. $\square$

#### Corollary 4.4

Let *X* be an idealized smooth log scheme over $\textbf{C}$. Then the following hold.

- For every morphism $f:Y\rightarrow X$ such that *Y* is idealized smooth, the pull-back functor
  $$\begin{aligned} f^*:\textrm{MIC}(X/\textbf{C})\rightarrow \textrm{MIC}(Y/\textbf{C}) \end{aligned}$$
  maps $$\textrm{MIC}_\textrm{reg}(X/\textbf{C})$$ into $$\textrm{MIC}_\textrm{reg}(Y/\textbf{C})$$.
- If $f:Y\rightarrow X$ is a log dominant (Definition 2.14) morphism of idealized smooth log schemes over $\textbf{C}$ and *E* is an object of $\textrm{MIC}(X/\textbf{C})$ such that $f^* E$ is regular, then *E* is regular as well.

#### Proof

The first assertion follows from characterization (b) in Lemma 4.3, and the second one from characterization (c). $\square$

#### Proposition 4.5

Let *X* be an idealized smooth log scheme over $\textbf{C}$. Then, the category $\textrm{MIC}_\textrm{reg}(X/\textbf{C})$ is an abelian subcategory of $\textrm{MIC}(X/\textbf{C})$ stable under direct sums, tensor products, exterior and symmetric powers, duals, internal Hom, subobjects, quotients, and extensions.

#### Proof

We will use the corresponding assertions about $\textrm{MIC}_\textrm{reg}(\underline{X}^\#/\textbf{C})$, see [6, Chap. II, Proposition 4.6].

Tensor operations. Let *E* and *F* be regular. Since the functor $\varepsilon ^\circledast _\textrm{univ} \pi ^*$ is monoidal, i.e.

$$\begin{aligned} \varepsilon ^\circledast _\textrm{univ} (\pi ^*(E\otimes F)) \simeq \varepsilon ^\circledast _\textrm{univ} (\pi ^* E) \otimes \varepsilon ^\circledast _\textrm{univ} (\pi ^* F), \end{aligned}$$

the assertion follows from the corresponding assertion about $\textrm{MIC}_\textrm{reg}(\underline{X}^\#/\textbf{C})$. Since exterior and symmetric powers are natural direct summands of tensor powers, we obtain the regularity of  as well.

Quotients. Since the functor $\varepsilon ^\circledast _\textrm{univ} \pi ^*$ is right exact (being the composition of the right exact $\pi ^*$ and the exact functor $\varepsilon ^\circledast _\textrm{univ}$), the assertion follows from the corresponding assertion about $\textrm{MIC}_\textrm{reg}(\underline{X}^\#/\textbf{C})$.

Extensions. Let $0\rightarrow E'\rightarrow E\rightarrow E''\rightarrow 0$ be a short exact sequence in $\textrm{MIC}(X/\textbf{C})$, with $E'$ and $E''$ regular. Applying the functor $\varepsilon ^\circledast _\textrm{univ} \pi ^*$, we obtain a right exact sequence in $\textrm{MIC}_\textrm{reg}(\underline{X}^\#/\textbf{C})$Therefore $\varepsilon ^\circledast _\textrm{univ} (\pi ^*E)$ is an extension of the regular $\varepsilon ^\circledast _\textrm{univ} (\pi ^*E'')$ by a quotient of the regular $\varepsilon ^\circledast _\textrm{univ} (\pi ^*E')$, and hence is regular.

Subobjects. Let $E'\rightarrow E$ be a monomorphism in $\textrm{MIC}(X/\textbf{C})$ where *E* is regular. We want to show that $E'$ is regular as well. The subtlety is that $\pi :X^\#\rightarrow X$ is not flat, and so $\pi ^*(E')\rightarrow \pi ^*(E)$ might not be injective.

Suppose first that *X* is hollow. In this case, $X^\#\rightarrow X^\flat =X$ is smooth, and the assertion follows from the classical case since $\varepsilon ^\circledast _\textrm{univ}(\pi ^* E') \rightarrow \varepsilon ^\circledast _\textrm{univ}(\pi ^* E')$ is injective.

The question is étale local on $\underline{X}$, so we may assume *X* is affine admits a strict étale map $X\rightarrow \textbf{A}_{P,K}$. In fact, by Lemma 2.2 we may reduce to the case $K=\varnothing$. For a face *F* of *P*, let $U_F$ be the preimage of $\textbf{A}_{P_F}$ in *X* and let $X_F$ be the preimage of $\textbf{A}_{F^\textrm{gp}}$ embedded as in (2.1), so that

$$\begin{aligned} X^\flat = \textbf{A}_P^\flat \times _{\textbf{A}_P} X \simeq \bigsqcup _F X_F. \end{aligned}$$

We need to show that the pull-back of $E'$ to every $X_F$ is regular. Fix a face $F\subseteq P$ and let $Z = X_F$ be the corresponding stratum. Since $X_F\subseteq U_F$, we may replace *X* with $U_F$ and hence assume *Z* is closed in *X*, cut out by an ideal . For $n\ge 0$, we let $Z^{(n)}$ be the closed subscheme cut out by the ideal .

As in the proof of Proposition 2.15, by the Artin–Rees lemma there exists a $k\ge 0$ and a surjection

$$\begin{aligned} \textrm{im}(E'\rightarrow E|_{Z^{(k)}}) \longrightarrow E'|_Z. \end{aligned}$$

We may then replace *X* with $Z^{(k)}$ and $E'$ with $\textrm{im}(E'\rightarrow E|_{Z^{(k)}})$. We have thus reduced to the case *X* with constant log structure.

We may reduce to the case  using the Snake Lemma applied to the diagramHowever,  itself is a quotient of . Since tensor products of regular objects are regular, we are finally reduced to showing that  is regular as an object of $\textrm{MIC}(X/\textbf{C})$.

To show that  is regular, we may assume that *X* admits a strict étale map $X\rightarrow \textbf{A}_{P,K}$ with  generated by $\sqrt{K} = P \setminus P^\times$. Thus  is the pullback of , and it suffices to treat $X=\textbf{A}_{P,K}$ and . Since $\overline{P}^\textrm{gp}$ is free, we may write $P=\overline{P}\times P^\times$. Then  is the pullback of the ideal generated by $\overline{P}{\setminus }\{0\}$ in $\textbf{A}_{\overline{P},\overline{K}}$ where $\overline{K}$ is the image of *K* in $\overline{P}$. This reduces us further to the case $X={{\,\textrm{Spec}\,}}(\overline{P}\rightarrow \textbf{C}[\overline{P}]/(\overline{K}))$, but then $\underline{X}$ is a point, and we conclude by Lemma 4.3(c).

Internal Hom. As in the proof above, we reduce to showing that if *E* and *F* are regular objects of $\textrm{MIC}(X/\textbf{C})$ and $Z\subseteq X$ is a closed log stratum, then $\underline{{{\,\textrm{Hom}\,}}}(E, F)|_Z$ is regular. If $X=Z$ (i.e. *X* is hollow), this is clear because $\varepsilon ^\circledast _\textrm{univ}(\pi ^*\underline{{{\,\textrm{Hom}\,}}}(E, F)) \simeq \underline{{{\,\textrm{Hom}\,}}}(\varepsilon ^\circledast _\textrm{univ}(\pi ^* E), \varepsilon ^\circledast _\textrm{univ}(\pi ^* F))$ by flatness of $\pi$. In general, by the Mittag–Leffler property (Lemma 4.6 below with $e=0$) there exists a $k\ge 0$ such that the natural map

$$\begin{aligned} \underline{{{\,\textrm{Hom}\,}}}(E, F)|_Z \longrightarrow \underline{{{\,\textrm{Hom}\,}}}(E|_{Z^{(k)}}, F|_{Z^{(k)}})|_Z \end{aligned}$$

is injective. Therefore we may replace *X* with $Z^{(k)}$. In this case, the short exact sequencesinduce exact sequencesand by induction on *j* we reduce to the case . In this case, , and we reduced to the case $X=Z$ already handled before. $\square$

The above proof used the following lemma from commutative algebra.

#### Lemma 4.6

Let *A* be a Noetherian ring, let $I\subseteq A$ be an ideal, and let *E* and *F* be finitely generated *A*-modules. For $n\ge 0$, set $A_n = A/I^{n+1}$, and for an *A*-module *M* we set $M_n = M\otimes _A A_n$. Then, for every $e\ge 0$ there exists a $k_e\ge e$ such that for every $k\ge k_e$ the morphism

$$\begin{aligned} {{\,\textrm{Hom}\,}}(E, F)_e \longrightarrow {{\,\textrm{Hom}\,}}(E_k, F_k)_e \end{aligned}$$

(induced by the restriction map $\pi :{{\,\textrm{Hom}\,}}(E, F)\rightarrow {{\,\textrm{Hom}\,}}(E_k, F_k)$) is injective.

#### Proof

Fix a presentation $A^m\rightarrow A^n \rightarrow E\rightarrow 0$, so that ${{\,\textrm{Hom}\,}}(E, F)$ is the cohomology module of

$$\begin{aligned} 0\longrightarrow F^n\longrightarrow F^m \end{aligned}$$

with $F^n\rightarrow F^m$ induced by the transpose of $A^m\rightarrow A^n$ (see [29, Tag 09BB]), and analogously ${{\,\textrm{Hom}\,}}(E_k, F_k)$ is the cohomology module of $0\rightarrow F_k^n\rightarrow F_k^m$. By the Mittag–Leffler property [29, Tag 0EGU], there exists a $k_e\ge e$ such that for $k\ge k_e$ there exists a $\psi _k$ making the triangle below commuteTensoring the above diagram with $A_e$ shows that ${{\,\textrm{Hom}\,}}(E, F)_e\rightarrow {{\,\textrm{Hom}\,}}(E_k, F_k)_e$ is a split injection. $\square$

#### Remark 4.7

Let *X* be an idealized smooth log scheme over $\textbf{C}$, let *E* be an object of $\textrm{MIC}(X/\textbf{C})$, and let $U\subseteq X$ be an open subset containing the associated points of *E*. Suppose that $E|_U$ is regular. Must *E* be regular as well?

The following provides a useful criterion for checking regularity using formal curve germs. For a space of the form $T={{\,\textrm{Spec}\,}}(P\xrightarrow {\varepsilon } \textbf{C}(\!(t)\!))$ where *P* is a sharp fs monoid and $\varepsilon (P\setminus \{0\}) = 0$, we write $\Omega ^1_{T/\textbf{C}}$ for the module of continuous log differentials. We have

$$\begin{aligned} \Omega ^1_{T/\textbf{C}} = \textbf{C}(\!(t)\!)d\log (t)\oplus (P^\textrm{gp}\otimes _\textbf{Z}\textbf{C}(\!(t)\!)). \end{aligned}$$

We define $\textrm{MIC}(T/\textbf{C})$ in the obvious way, and “projection onto $\textbf{C}(\!(t)\!)d\log (t)$” defines a functor

$$\begin{aligned} \varepsilon ^\circledast :\textrm{MIC}(T/\textbf{C})\longrightarrow \textrm{MIC}(\textbf{C}(\!(t)\!)/\textbf{C}) \end{aligned}$$

to the category of finite dimensional vector spaces over $\textbf{C}(\!(t)\!)$ endowed with an action of $t\frac{d}{dt}$ satisfying the Leibniz rule. As in [6, Chap. II, Définition 1.11], we say that an object *E* of $\textrm{MIC}(\textbf{C}(\!(t)\!)/\textbf{C})$ is *regular* if it admits a $\textbf{C}[\![t]\!]$-lattice stable under the action of $t\frac{d}{dt}$. For an idealized smooth log scheme *X* over $\textbf{C}$, a map $\gamma :T\rightarrow X$ induces a functor $\gamma ^*:\textrm{MIC}(X/\textbf{C})\rightarrow \textrm{MIC}(T/\textbf{C})$.

For $T={{\,\textrm{Spec}\,}}(P\xrightarrow {\varepsilon } \textbf{C}(\!(t)\!))$ as above and *X* an idealized smooth log scheme over $\textbf{C}$, let us say that a map $\gamma :T\rightarrow X$ is *algebraic* if it factors as $T\rightarrow C\rightarrow X$ where $C\hookrightarrow \overline{C}$ is a good embedding of idealized smooth log schemes of dimension one over $\textbf{C}$ with $C = \overline{C} {\setminus } \{y\}$, where *t* is a local parameter of  (identifying the fraction field of the completion of  with $\textbf{C}(\!(t)\!)$), and where  sending (*p*, *n*) to 0 if $p\ne 0$ and (0, *n*) to $t^n$ is a local chart at $\overline{C}$, and where $T\rightarrow C$ is induced by these data.

#### Proposition 4.8

Let *X* be an idealized smooth log scheme over $\textbf{C}$. An object *E* of $\textrm{MIC}(X/\textbf{C})$ is regular if and only if for every sharp fs monoid *P* and every strict morphism

$$\begin{aligned} f:{{\,\textrm{Spec}\,}}(P\xrightarrow {\varepsilon } \textbf{C}(\!(t)\!)) \longrightarrow X, \end{aligned}$$

the object $\varepsilon ^\circledast (f^* E)$ of $\textrm{MIC}(\textbf{C}(\!(t)\!)/\textbf{C})$ is regular. Moreover, it suffices to check this condition on algebraic maps *f*.

#### Proof

Let $E\in \textrm{MIC}(X/\textbf{C})$. By definition, *E* is regular if and only if the connection $\varepsilon ^\circledast _\textrm{univ} (\pi ^* E)$ on $\underline{X}^\#$ is regular. By [6, Chap. II, Théorème 4.1], this holds if and only if for every morphism

$$\begin{aligned} g:{{\,\textrm{Spec}\,}}\textbf{C}(\!(s)\!) \longrightarrow \underline{X}^\#, \end{aligned}$$

the pull-back $g^*(\varepsilon ^\circledast _\textrm{univ} (\pi ^* E))$ is regular, and it suffices to consider such germs which are “algebraic” in our sense. By the definition of $X^\#$, such a map corresponds to a morphism $f:{{\,\textrm{Spec}\,}}\textbf{C}(\!(s)\!)\rightarrow \underline{X}$ and a splitting $\varepsilon$ of . Moreover, we have

$$\begin{aligned} g^*(\varepsilon ^\circledast _\textrm{univ} (\pi ^* E)) \simeq \varepsilon ^\circledast (f^* E). \end{aligned}$$

Let $h:{{\,\textrm{Spec}\,}}\textbf{C}(\!(t)\!)\rightarrow {{\,\textrm{Spec}\,}}\textbf{C}(\!(s)\!)$ be a finite extension such that  is constant, so that  admits a global chart. Then $\varepsilon ^\circledast (f^* E)$ is regular if and only if $h^*(\varepsilon ^\circledast (f^* E))$ is. $\square$

#### Proposition 4.9

Let *X* be an idealized smooth log scheme over $\textbf{C}$ and let

$$\begin{aligned} f:{{\,\textrm{Spec}\,}}(P\xrightarrow {\varepsilon } \textbf{C}(\!(t)\!)) \longrightarrow X, \end{aligned}$$

be a strict morphism, where *P* is a sharp fs monoid. If the underlying map of schemes extends to ${{\,\textrm{Spec}\,}}\textbf{C}[\![t]\!]$, then for every $E\in \textrm{MIC}(X/\textbf{C})$ the object $\varepsilon ^\circledast (f^* E)$ of $\textrm{MIC}(\textbf{C}(\!(t)\!)/\textbf{C})$ is regular.

#### Proof

Let ${{\,\textrm{Spec}\,}}(\textbf{C}[\![t]\!])\rightarrow X$ be a morphism of schemes extending $\underline{f}$. We choose a chart for the pull-back log structure given by an fs monoid *Q* (it exists because $\textbf{C}[\![t]\!]$ is strictly local). This way we have obtained a strict morphism

$$\begin{aligned} \overline{f} :\Delta = {{\,\textrm{Spec}\,}}(Q\rightarrow \textbf{C}[\![t]\!]) \longrightarrow X \end{aligned}$$

extending *f*. The given splitting $\varepsilon$ corresponds to an isomorphism of log schemes4.1We denote by $F\subseteq Q$ the preimage of $\textbf{C}[\![t]\!]\setminus \{0\}$, which is a face of *Q*. Note that $P \simeq Q/F$.

By Lemma 4.2, we are allowed to change the splitting $\varepsilon$. We do this in the following way. Since $P^\textrm{gp}= Q^\textrm{gp}/F^\textrm{gp}$ is free, the quotient map $\rho :Q^\textrm{gp}\rightarrow P^\textrm{gp}$ admits a splitting $\sigma :P^\textrm{gp}\rightarrow Q^\textrm{gp}$. We write $\pi :Q^\textrm{gp}\rightarrow F^\textrm{gp}$ for the corresponding projection, $\pi = \textrm{id} - \sigma \circ \rho$. Consider the mapdefined in the following way. Since $\rho ^{-1}(P) = Q_F = Q - F$, for $p\in P$ we may write $\sigma (p) = q - f$ for $q\in Q$ and $f\in F$. We define

$$\begin{aligned} \varepsilon (p) = [(q, \alpha (f)^{-1})] \end{aligned}$$

which is easily seen to depend only on *p* and not on the choice of *q* and *f*. The map $\varepsilon$ induces a map of prelog rings $(\varepsilon , \textrm{id}):(P\rightarrow \textbf{C}(\!(t)\!))\rightarrow (Q\rightarrow \textbf{C}(\!(t)\!))$ and hence a map of log schemes as in (4.1), which is an isomorphism as the induced map on the stalks of  is the isomorphism .

We claim that it is enough to construct a functor $\overline{\varepsilon }^\circledast$ fitting inside a commutative (up to a natural transformation) square4.2where the vertical maps are induced by the appropriate strict open immersions. Indeed, the existence of such a diagram implies that

$$\begin{aligned} \varepsilon ^\circledast (f^* E) = \varepsilon ^\circledast (j^* \overline{f} ^* E) \simeq j^* \overline{\varepsilon }^\circledast (\overline{f} ^* E) \quad \in \quad \textrm{MIC}({{\,\textrm{Spec}\,}}(\textbf{C}(\!(t)\!))/\textbf{C}) \end{aligned}$$

admits an extension $\overline{\varepsilon }^\circledast (\overline{f} ^* E) \in \textrm{MIC}({{\,\textrm{Spec}\,}}(\textbf{N}\rightarrow \textbf{C}[\![t]\!])/\textbf{C})$ to a log connection, and therefore is regular.

In order to define the functor $\overline{\varepsilon }^\circledast$ in (4.2), we first define a map of $\textbf{C}[\![t]\!]$-modules

$$\begin{aligned} \overline{\varepsilon }^\circledast :\Omega ^1_{\Delta /\textbf{C}}\longrightarrow \Omega ^1_{(\textbf{N}\rightarrow \textbf{C}[\![t]\!])/\textbf{C}} = \textbf{C}[\![t]\!]\cdot d\log (t) \end{aligned}$$

by the formulas

$$\begin{aligned} \overline{\varepsilon }^\circledast (d\log q) = d\log \alpha (\pi (q)), \qquad \overline{\varepsilon }^\circledast (dt) = dt. \end{aligned}$$

The first formula makes sense since $\pi (q)\in F^\textrm{gp}$ implies $\alpha (\pi (q))\ne 0$ (here we are implicitly extending the map $\alpha :F\rightarrow \textbf{C}(\!(t)\!)^\times$ to $F^\textrm{gp}$), so that $d\log \alpha (\pi (q))$ is a section of $\Omega ^1_{(\textbf{N}\rightarrow \textbf{C}[\![t]\!])/\textbf{C}}$. For the two formulas to be compatible, we need to check the “log derivation” formula

$$\begin{aligned} \alpha (q) \cdot \overline{\varepsilon }^\circledast (d\log q) = d\alpha (q). \end{aligned}$$

For $q\notin F$, this reads $0=0$, while for $q\in F$ we have $\pi (q) = q$, so that $\overline{\varepsilon }^\circledast (d\log q) = d\log \alpha (q) = \alpha (q)^{-1} d\alpha (q)$. Thus the formula is satisfied in either case.

We define the functor $\overline{\varepsilon }^\circledast$ in the natural way

$$\begin{aligned} (E, \nabla )\quad \mapsto \quad (E, \quad E\xrightarrow {\nabla } E\otimes \Omega ^1_{\Delta /\textbf{C}} \xrightarrow {1\otimes \overline{\varepsilon }^\circledast } E\otimes \Omega ^1_{(\textbf{N}\rightarrow \textbf{C}[\![t]\!])/\textbf{C}}). \end{aligned}$$

It is trivial to check that the result of this is a connection (integrability is automatic since $\Omega ^2_{(\textbf{N}\rightarrow \textbf{C}[\![t]\!])/\textbf{C}}=0$), and functoriality is also clear.

Finally, the commutativity of (4.2) follows from the commutativity of the diagramWe check its commutativity on the generators $\{dt\}\cup \{d\log q\,:\,q\in Q\}$ of $\Omega ^1_{\Delta /\textbf{C}}$. The generator *dt* is mapped to *dt* in the three other corners of the square. For the generator $d\log q$, we first express $j^*(d\log q)$ using the generators $\{d\log p:\, p\in P\}$. Since

$$\begin{aligned} \varepsilon (\rho (q)) = [(q, \alpha (q - \sigma (\rho (q)))] = [(q, \alpha (\pi (q)))], \end{aligned}$$

we have

$$\begin{aligned} d\log q = d\log \alpha (\pi (q)) + d\log (\rho (q)), \end{aligned}$$

and hence (since $\varepsilon ^\circledast (d\log p) = 0$ for $p\in P$),

$$\begin{aligned} \varepsilon ^\circledast (j^* d\log q) = d\log \alpha (\pi (q)) = j^* d\log \alpha (\pi (q)) = j^* \overline{\varepsilon }^\circledast (d\log q). \end{aligned}$$

$$\square $$

Propositions 4.8 and 4.9 together imply:

#### Corollary 4.10

Let *X* be an idealized smooth log scheme over $\textbf{C}$ whose underlying scheme $\underline{X}$ is proper over $\textbf{C}$. Then $\textrm{MIC}_\textrm{reg}(X/\textbf{C}) = \textrm{MIC}(X/\textbf{C})$.

#### Proof

By the valuative criterion of properness, every morphism of ${{\,\textrm{Spec}\,}}(\textbf{C}(\!(t)\!))\rightarrow X$ of $\textbf{C}$-schemes extends uniquely to a morphism ${{\,\textrm{Spec}\,}}(\textbf{C}[\![t]\!])\rightarrow X$. Thus the assertion of Proposition 4.9 is satisfied. It remains to apply Proposition 4.8. $\square$

Finally, we turn to some basic cohomology comparison results in the case *X* has locally constant log structure.

#### Lemma 4.11

Let *X* be a hollow idealized smooth log scheme over $\textbf{C}$ and let *E* be an object of $\textrm{MIC}_\textrm{reg}(X/\textbf{C})$. Then, $H^*_\textrm{dR}(X, E) \simeq H^*_\textrm{dR}(X_\textrm{an}, E_\textrm{an})$.

#### Proof

We may assume that  is constant and that *X* admits a splitting $\varepsilon$. By Proposition 3.7, we have a map of spectral sequencesSince  is constant, each  is an object of $\textrm{MIC}_\textrm{reg}(\underline{X}/\textbf{C})$. Therefore by the classical comparison theorem [6, Chap. II, Théorème 6.2], the maps $\alpha _{ij}$ are isomorphisms, and hence so is $\alpha$. $\square$

#### Corollary 4.12

For *X* with locally constant log structure and *E* regular, we have $H^*_\textrm{dR}(X, E) \simeq H^*_\textrm{dR}(X_\textrm{an}, E_\textrm{an})$.

#### Proof

Let  be the ideal of $X_\textrm{red}$ in *X*. We prove by induction on $n\ge 0$ that if  then the assertion holds. If $n=0$, then *E* is a connection on $X_\textrm{red}$ which is hollow and we conclude by Lemma 4.11. For the induction step, we note that in the the extension  the assertion holds for both  and  by the induction assumption. Using the five lemma, we conclude that it holds for *E* as well. $\square$

#### Remark 4.13

The strategy of using $X^\#$ and the functor $\varepsilon ^\circledast _\textrm{univ}(\pi ^*(-))$ to study $\textrm{MIC}(X/\textbf{C})$ applies similarly to categories of coherent log *D*-modules on *X* [17, 18] (see Remark 3.6.5). For example, if $Z\subseteq X$ is a closed subset, then its log dimension ${\text {logdim}}(Z)$ introduced in [18] equals the dimension of $\pi ^{-1}(Z)\subseteq X^\#$. Moreover, using Lemma 3.10 one can show that an object *M* of  is holonomic if and only if the object $\varepsilon ^\circledast _\textrm{univ}(\pi ^*(M))$ of  is holonomic in the classical sense. It seems natural to call a coherent -module *M* *regular holonomic* if $\varepsilon ^\circledast _\textrm{univ}(\pi ^*(M))$ is regular holonomic. That said, we did not investigate this notion beyond the case of -coherent -modules i.e. objects of $\textrm{MIC}(X/\textbf{C})$.

### The Existence Theorem

#### Theorem 4.14

(Existence theorem) Let *X* be an idealized smooth log scheme over $\textbf{C}$. Then the composite functor

$$\begin{aligned} \textrm{MIC}_\textrm{reg}(X/\textbf{C}) \hookrightarrow \textrm{MIC}(X/\textbf{C}) \xrightarrow {E\mapsto E_\textrm{an}} \textrm{MIC}(X_\textrm{an}/\textbf{C}) \end{aligned}$$

is an equivalence of categories.

#### Proof

We first show that the functor is fully faithful. For objects *E* and *F* of $\textrm{MIC}_\textrm{reg}(X/\textbf{C})$ consider the object $H = \underline{{{\,\textrm{Hom}\,}}}(E, F)$. It is again regular by Proposition 4.5, and we have ${{\,\textrm{Hom}\,}}(E, F) = H^0_\textrm{dR}(X, H)$ and ${{\,\textrm{Hom}\,}}(E_\textrm{an}, F_\textrm{an}) = H^0_\textrm{dR}(X_\textrm{an}, H_\textrm{an})$. So we need to show that for a regular object *H* we have $H^0_\textrm{dR}(X, H)\simeq H^0_\textrm{dR}(X_\textrm{an}, H_\textrm{an})$. To this end, Proposition 2.15 applied to the log stratification of *X* allows us to reduce to the case when the log structure on *X* is locally constant. In this case we apply Corollary 4.12 to conclude.

It remains to show essential surjectivity. By Corollary 4.4(b), the assertion is étale local on $\underline{X}$. Therefore by Theorem 2.5 we may assume that there exists a good compactification $X\hookrightarrow \overline{X}$. Consider the commutative square of categories and functorsThe top functor is an equivalence by Lemma 4.10 and GAGA. The right functor is essentially surjective by Proposition 3.17. So the bottom functor is essentially surjective. $\square$

As a byproduct of the proof, we obtain another familiar-looking characterization of regular connections.

#### Corollary 4.15

(Regularity = log extendability) Let *X* be an idealized smooth log scheme over $\textbf{C}$ and let *E* be an object of $\textrm{MIC}(X/\textbf{C})$. The following are equivalent:

- *E* is regular,
- for every strict étale $f:U\rightarrow X$ and every good compactification $j:U\hookrightarrow \overline{U}$, there exists an object $\overline{E}$ of $\textrm{MIC}(\overline{U}/\textbf{C})$ such that $j^*\overline{E} \simeq f^* E$,
- there exists a strict étale cover $\{f_i:U_i\rightarrow X\}_{i\in I}$, good compactifications $j_i:U_i \rightarrow \overline{U}_i$, and objects $\overline{E}_i$ of $\textrm{MIC}(\overline{U}_i/\textbf{C})$ such that $j^*_i\overline{E}_i \simeq f_i^* E$ for every $i\in I$.

#### Remark 4.16

Is there a characterization of regular logarithmic connections in terms of growth conditions akin to [6, Chap. II, Théorème 4.1(iii)]?

### The Comparison Theorem

#### Theorem 4.17

(Comparison theorem) Let *X* be an idealized smooth log scheme over $\textbf{C}$ and let *E* be an object of $\textrm{MIC}_\textrm{reg}(X/\textbf{C})$. Then $H^*_\textrm{dR}(X, E)\simeq H^*_\textrm{dR}(X_\textrm{an}, E_\textrm{an})$.

The proof will rely on slight variants of two results of Ogus, adapted to our notion of a good compactification.

#### Theorem 4.18

(Variant of [27, Theorem 3.4.9]) Let $j:X\hookrightarrow \overline{X}$ be a good embedding of idealized smooth log complex analytic spaces. Let *E* be an object of $\textrm{MIC}(\overline{X}/\textbf{C})$, and suppose that the following two conditions are satisfied

1. For every $x\in X$, the set $R_x$ of exponents at infinity (§3.3) of *E* at *x* satisfies .
2. $$E\rightarrow j_* j^* E$$ is injective.

Then $$H^*_\textrm{dR}(\overline{X}, E)\simeq H^*_\textrm{dR}(X, j^* E)$$.

#### Proof

As in [27], we may reduce to an analogous statement about monoids. Let *P* be a sharp fs monoid and let $P\rightarrow P'$ be a localization with respect to a face $F\subseteq P$. Let *V* be an object of *L*(*P*) (Example 3.14) and let $V' = V\otimes _{\textbf{C}[P]} \textbf{C}[P']$. Suppose that (1) $V\rightarrow V'$ is injective (i.e. *V* is *F*-torsion free) and that (2) the $F^\textrm{gp}$-graded $\textbf{C}[F]$-submodule ${\tilde{V}} = \bigoplus _{\lambda \in F^\textrm{gp}} V_\lambda$ is generated by elements lying in gradings in $-F\subseteq F^\textrm{gp}$. The statement we need (itself a variant of [27, Corollary 1.4.6]) is that the map on group cohomology

$$\begin{aligned} H^*(\pi _1(P), V_0) \longrightarrow H^*(\pi _1(P), V'_0) \end{aligned}$$

is an isomorphism. As in the proof of [27, Corollary 1.4.6], it suffices to observe that in fact $V_0=V'_0$ (variant of [27, Corollary 1.1.3]). By assumption (1), the map $V_0\rightarrow V'_0$ is injective, so we need to prove surjectivity. Let *f* be a generator of *F* as a face, so that for every $x\in F$ there exists an $y\in F$ and an integer $n\ge 0$ such that $x+y=nf$. Then $V' = V[1/f]$ and writing the localization as a colimit we have

$$\begin{aligned} V'_0 = \varinjlim _{n}\, V_{nf}. \end{aligned}$$

Let $v'\in V'_0$, and represent it by an element $v'\in V_{nf}$ for some $n\ge 0$. By assumption (2) there exist homogeneous generators $v_1, \ldots , v_s$ of $\tilde{V}$ with $v_i\in V_{-f_i}$ for some $f_i\in F$. Thus, we can write $v' = \sum g_i v_i$ with $g_i\in \textbf{C}[F]_{nf+f_i}$. We may write $g_i = \alpha _i f^n f_i$ (note the change of notation from additive to multiplicative when passing from monoids to rings) where $\alpha _i\in \textbf{C}$, and set $v = \sum \alpha _i f_i v_i \in V_0$. Then $v' = f^n v$, and so $v'$ and *v* have the same image in $V'_0$. $\square$

#### Theorem 4.19

(Variant of [27, Theorem 3.4.10]) Let $j:X\hookrightarrow \overline{X}$ be a good embedding of idealized smooth log schemes over $\textbf{C}$ and let *E* be an object of $\textrm{MIC}(\overline{X}_\textrm{an}/\textbf{C})$. Denote by $j_* j_m^*$ the functor of meromorphic sections as in [6, Chap. II, 3.12]. Then the inclusion

$$\begin{aligned} j_* j_m^* (E\otimes \Omega ^\bullet _{\overline{X}/\textbf{C}}) \longrightarrow j_* j^* (E\otimes \Omega ^\bullet _{\overline{X}/\textbf{C}}) \end{aligned}$$

is a quasi-isomorphism.

#### Proof

Repeat the original proof, replacing the use of [27, Theorem 3.4.9] with Theorem 4.18. $\square$

#### Proof of Theorem 4.17

By cohomological descent, the statement is étale local on $\underline{X}$, so we may assume that *X* admits a good compactification $j:X\rightarrow \overline{X}$. By Corollary 4.15 of the Existence Theorem, *E* extends to an object $\overline{E}$ of $\textrm{MIC}_\textrm{reg}(\overline{X}/\textbf{C}) = \textrm{MIC}(X/\textbf{C})$. Since *j* is affine and $j_\textrm{an}$ is Stein, arguing as in [6, Chap. II, §6.6] we obtain

$$\begin{aligned} H^*_\textrm{dR}(X, E)\simeq &   H^*(\overline{X}_\textrm{an}, j_* j_m^* (E\otimes \Omega ^\bullet _{\overline{X}/\textbf{C}})) \quad \text {and}\quad H^*_\textrm{dR}(X_\textrm{an}, E_\textrm{an})\\\simeq &   H^*(\overline{X}_\textrm{an}, j_* j^* (E\otimes \Omega ^\bullet _{\overline{X}/\textbf{C}})). \end{aligned}$$

It remains to invoke Theorem 4.19 to conclude. $\square$

### The Regularity Theorem

We recall the Katz–Oda construction of the Gauss–Manin connection [16] in the relevant context (see e.g. [12]). Let $f:Y\rightarrow X$ be a proper smooth morphism of idealized smooth log schemes over $\textbf{C}$. We have the short exact sequencefrom which we obtain a decreasing filtration on $\Omega ^q_{Y/\textbf{C}} = \wedge ^q \Omega ^1_{Y/\textbf{C}}$

$$\begin{aligned} K^r \Omega ^q_{Y/\textbf{C}} = \textrm{im}\left( f^*\Omega ^{r}_{X/\textbf{C}}\otimes \Omega ^{q-r}_{Y/\textbf{C}}\rightarrow \Omega ^q_{Y/\textbf{C}}\right) \end{aligned}$$

satisfying

$$\begin{aligned} \textrm{gr}^r \Omega ^q_{Y/\textbf{C}} = K^r\Omega ^q_{Y/\textbf{C}}/K^{r+1}\Omega ^q_{Y/\textbf{C}} \simeq f^*\Omega ^{r}_{X/\textbf{C}}\otimes \Omega ^{q-r}_{Y/X}. \end{aligned}$$

Let *E* be an object of $\textrm{MIC}(Y/\textbf{C})$. Then for every $r\ge 0$, $E\otimes K_r\Omega ^\bullet _{Y/\textbf{C}}$ is a subcomplex of $E\otimes \Omega ^\bullet _{Y/\textbf{C}}$ and

$$\begin{aligned} {\text {gr}}^r(E\otimes \Omega ^\bullet _{Y/\textbf{C}}) \simeq f^*\Omega ^r_{X/\textbf{C}}\otimes (E\otimes \Omega ^{\bullet -r}_{Y/X}). \end{aligned}$$

In particular we have a short exact sequence of complexesApplying $Rf_*$ and the projection formula, we obtain as the connecting homomorphism

$$\begin{aligned} \nabla _\textrm{GM}:R^n f_* (E\otimes \Omega ^\bullet _{Y/X}) \longrightarrow R^n f_* (E\otimes \Omega ^\bullet _{Y/X}) \otimes \Omega ^1_{X/\textbf{C}}. \end{aligned}$$

As in the classical case, one shows that this connection is integrable, which makes $R^n f_* (E\otimes \Omega ^\bullet _{Y/X})$ into an object of $\textrm{MIC}(X/\textbf{C})$.

The analogous construction applies to smooth proper morphisms of idealized smooth log complex analytic spaces, in particular to $f_\textrm{an}$. This way, we obtain an object $R^n f_\mathrm{an,*} (E_\textrm{an}\otimes \Omega ^\bullet _{Y_\textrm{an}/X_\textrm{an}})$ of $\textrm{MIC}(X_\textrm{an}/\textbf{C})$. We have a comparison morphism

4.3 $$\begin{aligned} (R^n f_* (E\otimes \Omega ^\bullet _{Y/X}))_\textrm{an} \longrightarrow R^n f_\mathrm{an,*} (E_\textrm{an}\otimes \Omega ^\bullet _{Y_\textrm{an}/X_\textrm{an}}) \end{aligned}$$

which is an isomorphism because *f* is proper.

#### Remark 4.20

The main result of the PhD thesis of Aaron Gray [9] (unpublished) asserts that under certain assumptions, the formation of higher direct images is compatible with Ogus’ Riemann–Hilbert correspondence  (Theorem 3.12). More precisely, for a proper and smooth morphism of smooth log complex analytic spaces $f:Y\rightarrow X$ such that the map  is injective and its cokernel is torsion-free, there is a natural isomorphism between the two compositions in the square4.4where the functor $R^n f^\Lambda _*$ is constructed as follows. Recall (Definition 3.11) that an object *V* of *L*(*Y*) is a $\Lambda _Y$-graded $\textbf{C}_Y^\textrm{log}$-module satisfying certain conditions. Via $\textbf{C}_X^\textrm{log}\rightarrow f^\textrm{log}_* \textbf{C}_Y^\textrm{log}$, the sheaf $R^n f^\textrm{log}_* V$ is a $f_* \Lambda _Y$-graded $\textbf{C}_X^\textrm{log}$-module. Using the map $\iota :\Lambda _X\rightarrow f^\textrm{log}_* \Lambda _Y$, we define the $\Lambda _X$-graded $\textbf{C}_X^\textrm{log}$-module $R^n f^\Lambda _* V$ by $(R^n f^\Lambda _* V)_\lambda = (R^n f^\textrm{log}_* V)_{\iota (\lambda )}$ for a local section $\lambda$ of $\Lambda _X$. Then $R^n f^\Lambda _* V$ is an object of *L*(*X*).

Recently, Gray’s results have been extended to log *D*-modules by Koppensteiner [17, Proposition 6.4].

The Regularity Theorem, stated below, asserts the regularity of $R^n f_* (E\otimes \Omega ^\bullet _{Y/X})$ assuming that *E* is regular. It is conditional on the following logarithmic variant of semistable reduction. We hope it is within reach of the current methods [2, 25, 30].

#### Conjecture 4.21

(Log semistable reduction over a hollow curve) Let *X* be a hollow idealized smooth log scheme over $\textbf{C}$ of dimension $\le 1$ and let $Y\rightarrow X$ be a smooth, proper, and exact morphism. Then, possibly after passing to a Kummer étale cover of *X*, there exists a commutative squarewhere $X\hookrightarrow \overline{X}$ and $Y\hookrightarrow \overline{Y}$ are good compactifications (Definition 2.3) and where $\overline{Y}\rightarrow \overline{X}$ is smooth.

The above conjecture is true if *X* has trivial log structure and if $\underline{Y}$ is smooth, in which case it follows from embedded resolution of singularities. This fact has been employed by Deligne in his proof of the regularity theorem [6, Chapitre II, Théorème 7.9].

#### Theorem 4.22

(Regularity theorem) Let $f:Y\rightarrow X$ be a proper, smooth and exact morphism of idealized smooth log schemes over $\textbf{C}$ and let *E* be an object of $\textrm{MIC}_\textrm{reg}(Y/\textbf{C})$. Assume that Conjecture 4.21 holds. Then $R^n f_*(E\otimes \Omega ^\bullet _{Y/X})$ are objects of $\textrm{MIC}_\textrm{reg}(X/\textbf{C})$ for all $n\ge 0$.

#### Proof

*Step 1—reduction to X with constant log structure.* Set $V^n = R^n f_*(E\otimes \Omega ^\bullet _{Y/X})$. We need to show that for every log stratum $Z\subseteq X$ (a connected component of $X^\flat$), the restrictions $V^n|_Z$ are regular. Since *Z* is locally closed in *X*, we may replace *X* with an affine open subset in which $Z\cap X$ is closed. Let  be the ideal of the closed immersion $Z\hookrightarrow X$, and let  be the *k*-th order thickening of *Z* and let

$$\begin{aligned} V^{n,k} = R^n (f|_{Z^{(k)}})_*(E|_{f^{-1}(Z^{(k)})}\otimes \Omega ^\bullet _{Y/X}). \end{aligned}$$

As in [10, §4.1], the inverse system of coherent -modules $V^{n,k}$ satisfies the Mittag–Leffler property, and if we set $R_k = \ker (V^n\rightarrow V^{n,k})$, then the submodules $\{R_k\}$ define the -adic topology on $V^n$. In particular, for *k* large enough we have , and hence a factorizationThus $V^n|_Z$ is a subquotient of $V^{n,k}$ and by Proposition 4.5 it suffices to show that $V^{n,k}$ is regular. We may therefore replace *X* with $Z^{(k)}$.

*Step 2—reduction to* *X* *hollow.* Let *X* have locally constant log structure and let $Z = X_\textrm{red}$ be the largest hollow subscheme of *X*, cut out by an ideal . Suppose that the assertion holds for $X=Z$. We show by induction on $m\ge 0$ that if *E* is annihilated by , then the higher direct images $R^n f_*(E\otimes \Omega ^\bullet _{Y/X})$ are regular for all $n\ge 0$. The base case $n=0$ corresponds to *E* supported on *Z*, which follows from the case $X=Z$. For the induction step, use the long exact sequence obtained by applying $R^n f_*((-)\otimes \Omega ^\bullet _{Y/X})$ to the short exact sequence in $\textrm{MIC}_\textrm{reg}(Y/\textbf{C})$*Step 3—ensuring base change.* Suppose that *X* is hollow. Since *X* is reduced, there is a dense open $U\subseteq X$ on which the sheaves $R^j f_* (E\otimes \Omega _{Y/X}^i)$ are locally free and (hence) with formation commuting with base change for all $i,j\ge 0$. From the spectral sequence

$$\begin{aligned} E_1^{ij}=R^j f_* (E\otimes \Omega _{Y/X}^i) \quad \Rightarrow \quad R^{i+j} f_*(E\otimes \Omega ^\bullet _{Y/X}), \end{aligned}$$

we deduce that the formation of $R^n f_*(E\otimes \Omega ^\bullet _{Y/X})$ commutes with base change along strict morphisms $X'\rightarrow X$ with $X'$ idealized smooth over $\textbf{C}$ such that $\textrm{im}(X'\rightarrow X)\subseteq U$. Recall that by Corollary 4.4(b) to prove the assertion we may replace *X* with a dense open subset. We may therefore assume that the base change property holds with $U=X$.

*Step 4—reduction to the case* *X* *being a curve.* Suppose that *X* is hollow and that the formation of the commutes with base change as above $R^n f_*(E\otimes \Omega ^\bullet _{Y/X})$. By the criterion of Proposition 4.8 it suffices to show that if $\gamma :C\rightarrow X$ is a strict map where *C* smooth curve, then $\gamma ^*(R^n f_*(E\otimes \Omega ^\bullet _{Y/X}))$ is regular for all $n\ge 0$. By the base change property, we have

$$\begin{aligned} \gamma ^*(R^n f_*(E\otimes \Omega ^\bullet _{Y/X})) \simeq R^n (f_C)_* (E_{Y_C}\otimes \Omega ^\bullet _{Y_C/C}). \end{aligned}$$

Therefore, if the assertion holds for all such $C\rightarrow X$, then it holds for *X*.

*Step 5—semistable reduction.* Suppose that *X* is a hollow curve. By Conjecture 4.21, after passing to a Kummer étale cover the map $Y\rightarrow X$ extends to a smooth morphism of good compactifications $\overline{f}:\overline{Y}\rightarrow \overline{X}$. By Corollary 4.15, *E* extends to an object $\overline{E}$ of $\textrm{MIC}_\textrm{reg}(\overline{Y}/\textbf{C})$. Since $Y=\overline{Y}\times _{\overline{X}} X$, we have $R^n f_*(E\otimes \Omega ^\bullet _{Y/X}) \simeq (R^n \overline{f}_*(\overline{E}\otimes \Omega ^\bullet _{\overline{Y}/\overline{X}})|_X$. Therefore $R^n f_*(E\otimes \Omega ^\bullet _{Y/X})$ extends to the compactification $\overline{X}$ and is therefore regular (Corollary 4.15 again). $\square$

#### Remark 4.23

In the situation of the classical Regularity Theorem, it is also true that the formation of $R^n f_* (E\otimes \Omega ^\bullet _{Y/X})$ commutes with base change [6, Chap. II, Proposition 6.14]. This is not true in general for proper smooth morphisms of smooth log schemes over $\textbf{C}$, as the following example shows.

Let $X=\textbf{A}_{\textbf{N}^2}$ and let $f:Y\rightarrow X$ be the blowup of the origin *P*. Let $D\subseteq Y$ be the exceptional divisor, and set . It inherits a logarithmic connection, as its dual  is a subobject of  in $\textrm{MIC}(Y/\textbf{C})$. As *f* is étale, we have  and . However, we have , which violates base change along the strict inclusion $P\hookrightarrow X$.

However, assuming that the map  is injective and its cokernel is torsion-free, we can use Gray’s results [9] (Remark 4.20) and proper base change for maps of topological spaces to show the base change property for $R^n f_* (E\otimes \Omega ^\bullet _{Y/X})$ along a strict map $g:X'\rightarrow X$ as follows. Since (4.3) is an isomorphism, it is enough to show the base change property in the analytic context, so changing notation from now on $Y\rightarrow X$ is a smooth proper morphism of idealized smooth complex analytic spaces satisfying the assumption on  and $g:X'\rightarrow X$ is a strict map of idealized smooth log complex analytic spaces. We set $Y' = Y\times _X X'$. As *g* is strict, the four squares marked $\square$ in the diagram below are cartesian squares of topological spaces.Moreover, we have $\textbf{C}_{Y'}^\textrm{log} = g_\textrm{log}^* \textbf{C}_Y^\textrm{log}$ and $\textbf{C}_{X'}^\textrm{log} = g_\textrm{log}^* \textbf{C}_X^\textrm{log}$ (as *X* and $X'$ are hollow). Therefore the functors $g^*:L(Y)\rightarrow L(Y')$ and $g^*:L(X)\rightarrow L(X')$ are just sheaf-theoretic pullback. By proper base change applied to $\tau _X f_\textrm{log}$, for an object *V* of *L*(*Y*) we have4.5Therefore for an object *E* of $\textrm{MIC}(Y/\textbf{C})$, the base change map factors into a chain of isomorphisms

## Connections and local systems

### Definition 5.1

An idealized smooth log scheme over $\textbf{C}$ or log complex analytic space *X* is *of nc (normal crossings) type* if the stalks of  are free monoids.

Let *X* be log complex analytic space of nc type. Analogously to the definition in §3.3, for an object *V* of *L*(*X*) and $x\in X$ we denote by  the set of exponents of *V* at *x* [27, Definition 2.1.1]. Picking an isomorphism , we may view $R_x$ as a subset of $\textbf{C}^{r_x}$, well defined up to coordinate permutation.

### Definition 5.2

Let *X* be a log complex analytic space of nc type and let $\sigma :\textbf{C}/\textbf{Z}\rightarrow \textbf{C}$ be a section of the projection $\textbf{C}\rightarrow \textbf{C}/\textbf{Z}$. We say that an object *V* of *L*(*X*) is $\sigma$*-adapted* if

1. for every $x\in X$, the set $R_x$ of exponents of *V* at *x* is contained in $\textrm{im}(\sigma )^{r_x}$, and
2. *V* is locally free as a module over $\textbf{C}_X^\textrm{log}$.

We denote by $L^\sigma (X)$ the full subcategory of *L*(*X*) consisting of $\sigma$-adapted objects.

On the space $X_\textrm{log}$, we have a surjective map of sheaves of ringssending the nonzero elements of  to zero. This defines a functor

$$\begin{aligned} V\mapsto \underline{V} = V\otimes _{\textbf{C}_X^\textrm{log}} \textbf{C}\end{aligned}$$

from the category *L*(*X*) to the category of log-constructible sheaves of $\textbf{C}$-vector spaces on $X_\textrm{log}$, sending locally free objects to locally constant sheaves.

### Theorem 5.3

Let *X* be a log complex analytic space of nc type and let $\sigma :\textbf{C}/\textbf{Z}\rightarrow \textbf{C}$ be a section of the projection $\textbf{C}\rightarrow \textbf{C}/\textbf{Z}$.

- The functor $V\mapsto \underline{V}$ induces an equivalence of categories
- If $\sigma (0)=0$, then for every object *V* of $L^\sigma (X)$ the map $V_0\hookrightarrow V\rightarrow \underline{V}$ induces an isomorphism

### Proof

We describe the inverse functor. Let *W* be a complex local system on $X^\textrm{log}$. We set $V = W\otimes _\textbf{C}\textbf{C}_X^\textrm{log}$. This is a locally free $\textbf{C}_X^\textrm{log}$-module satisfying conditions (1), (2), and (3) of Definition 3.11. In order to make it into an object of $L^\sigma (X)$, we need to endow it with a $\Lambda _X$-grading satisfying condition (4) of Definition 3.11 and (2) of Definition 5.2. Let $x\in X^\textrm{log}$, and pick an isomorphism . The stalk $W_x$ is a representation ofLet $W_x = \bigoplus W_{x,\chi }$ be the decomposition of $W_x$ into generalized eigenspaces, the direct sum taken over

$$\begin{aligned} \chi \in {{\,\textrm{Hom}\,}}(\pi _1(\tau ^{-1}(\tau (x)), x), \textbf{C}^\times ) = {{\,\textrm{Hom}\,}}(\textbf{Z}(1)^{r_x}, \textbf{C}^\times ) = (\textbf{C}/\textbf{Z})^{r_x}. \end{aligned}$$

Thus $V = \bigoplus _\chi V_\chi$ where $V_\chi = W_\chi \otimes _\textbf{C}\textbf{C}_X^\textrm{log}$. We have $\Lambda _{X,x} = \textbf{C}^{r_x}$, and we endow $V_\chi$ with the $\textbf{C}^{r_x}$-grading where $w\otimes 1$ has degree $\sigma (\chi )$ where $\sigma :(\textbf{C}/\textbf{Z})^{r_x}\rightarrow \textbf{C}^{r_x}$ is the map induced by $\sigma$. It is clear that this grading is independent of the choice of generators of , that it satisfies the two required conditions, and that the $\Lambda _{X,x}$-gradings on $V_x$ for all $x\in X^\textrm{log}$ give rise to a $\Lambda _{X}$-grading on *V*. Trivially, $\underline{V}=W$. Moreover, the object associated to $W=\underline{V}$ is *V*, as *V* is freely generated by $\bigoplus _{\lambda \in \textrm{im}(\sigma )^{r_x}} V_\lambda =W$ (cf. the proof of Lemma 3.18).

For the second assertion, it is enough to check that the map $R\tau _{X,*}V_0\rightarrow R\tau _{X,*}\underline{V}$ is a quasi-isomorphism. By proper base change, it is enough to show that for every $x\in X$ we havePick an isomorphism  and write $\underline{V} = \bigoplus _\chi \underline{V}_\chi$ as in the previous paragraph. Then, for a base point $x'\in \tau ^{-1}(x)$

$$\begin{aligned} H^*(\tau ^{-1}(x), \underline{V}_\chi ) = H^*(\textbf{Z}(1)^{r_x}, V_{\sigma (\chi ),x'}) = 0 \quad \text {for}\, \chi \ne 0 \end{aligned}$$

(see the last assertion of [27, Proposition 1.4.3]). Therefore

$$\begin{aligned} H^*(\tau ^{-1}(x), \underline{V}) = H^*(\tau ^{-1}(x), \underline{V}_0) = H^*(\tau ^{-1}(x), V_{\sigma (0)}) = H^*(\tau ^{-1}(x), V_0).\qquad \end{aligned}$$

$$\square $$

### Corollary 5.4

Let *X* be a log scheme of nc type over $\textbf{C}$ and let $\sigma :\textbf{C}/\textbf{Z}\rightarrow \textbf{C}$ be a section of the projection $\textbf{C}\rightarrow \textbf{C}/\textbf{Z}$. Denote by $\textrm{MIC}^\sigma _\textrm{reg}(X/\textbf{C})$ the full subcategory of $\textrm{MIC}_\textrm{reg}(X/\textbf{C})$ consisting of objects which are locally free (as -modules) and whose exponents belong to the image of $\sigma$.

- Then there is an equivalence of categories
- If $\sigma (0)=0$, then for every object *E* of $\textrm{MIC}^\sigma _\textrm{reg}(X/\textbf{C})$ we have

We warn the reader that neither of the functors described in Theorem 5.3 and Corollary 5.4 is compatible with tensor product (actually, the subcategories $L^\sigma (X)$ and $\textrm{MIC}^\sigma _\textrm{reg}(X/\textbf{C})$ are not closed under tensor product).

## Data Availability

The manuscript has no associated data.

## Footnotes

1 (Added during revision) See, however, the notion of “virtual morphisms” introduced by Howell [11] and studied in the recent preprint [7].

2 An earlier version of the article erroneously claimed that  holds for every good embedding.

3 In the case of formal schemes, replace *nilpotent* with *topologically nilpotent*. Indeed, in an adic ring the intersection of all open prime ideals is the set of topologically nilpotent elements.

4 If *F* is dualizable, then the data of an *F*-Higgs field on an object *E* is equivalent to the structure of an ${\text {Sym}}(F^\vee )$-module on *E*. As pointed out by Langer [20, §4, especially Remark 4.12] in general it is better to work with the category of ${\text {Sym}}(L)$-modules for an object *L* of —for example, unlike the category of *F*-Higgs objects in our sense, it is always an abelian category. Fortunately in our setting the object *F* will always be dualizable.

5 In [27] this theorem is stated for analytifications of idealized smooth log schemes over $\textbf{C}$, but the proof does not make use of that assumption.
